# Large-Scale Prospective Virtual Screening Using a Co-Folding-Rescoring Workflow for NPM1-TYK2 Inhibitor Discovery

**DOI:** 10.3390/ijms27219864

**Published:** 2026-11-03

**Authors:** Weixin Tian, Xujun Zhang, Odin Zhang, Rubi Hu, Yuanyi Ye, Shicheng Chen, Jiale Zheng, Hui Zhang, Xin Chai, Wanglin Qu, Huating Wang, Dan Li, Yu Kang, Tingjun Hou, Peichen Pan

**Affiliations:** 1 School of Pharmacy, Zhejiang University, Hangzhou 310058, China; 2 Department of Computer Science and Engineering, The Chinese University of Hong Kong, Shatin, Hong Kong; 3 Zhongguancun Academy, Beijing 100094, China; 4 Zhejiang Provincial Key Laboratory for Intelligent Drug Discovery and Development, Jinhua Institute of Zhejiang University, Jinhua 321099, China

**Keywords:** virtual screening, artificial intelligence, drug design, TYK2 inhibitors, NPM1–TYK2, protein–ligand co-folding, AlphaFold3, molecular docking, enrichment factor, hit discovery

## Abstract

Conventional large-scale structure-based virtual screening typically relies on fixed-receptor docking, in which ligand poses are sampled against a predefined receptor conformation. Protein-ligand co-folding models can instead generate candidate complex conformations by jointly modeling ligand placement and local receptor-ligand structural adaptation. Although recent benchmark studies have demonstrated the promise of AlphaFold3-like approaches for retrospective virtual screening, their practical application to target-specific, large-scale prospective screening with experimental validation remains limited. Here, we developed and prospectively applied a co-folding-rescoring workflow for NPM1-TYK2 inhibitor discovery. Using an in-house set of 4608 compounds experimentally annotated in Ba/F3-NPM1-TYK2 cells, we evaluated 13 pipelines combining AlphaFold 3, Protenix, Glide, or AutoDock Vina pose generation with scoring functions or confidence metrics. AlphaFold 3 followed by AutoDock Vina rescoring achieved the numerically highest BEDROC value, closely comparable to Protenix + Vina, and was selected for screening a commercial library of more than 300,000 compounds. AF3-generated complex conformations were subsequently rescored with Vina and prioritized by physicochemical assessment, clustering, and binding-mode inspection. Of 30 compounds tested experimentally, FV13 was identified as a cell-active primary hit, with a TYK2 kinase IC_50_ of 21.70 μM and a Ba/F3-NPM1-TYK2 cellular IC_50_ of 17.07 μM. Subsequent analog exploration yielded TX14, exhibiting an approximately 9.4-fold improvement in biochemical TYK2 inhibitory potency (IC_50_ = 2.31 μM). Additional selectivity profiling and Western blot analyses further characterized these compounds and indicated attenuation of downstream STAT5 phosphorylation at higher concentrations. Together, these findings demonstrate the feasibility of integrating co-folding, empirical rescoring, and target-specific experimental data for large-scale prospective hit discovery.

## 1. Introduction

Oncogenic kinase fusions constitute an important class of cancer-driving genomic alterations in which chromosomal rearrangements generate aberrantly activated signaling proteins that promote malignant transformation and tumor-cell survival [1,2]. These fusion kinases commonly retain an intact catalytic domain while acquiring abnormal regulatory, localization, or oligomerization features from the fusion partner, leading to ligand-independent activation and sustained downstream signaling [1,2,3]. From a therapeutic perspective, such fusion events can generate strong tumor dependencies, making the retained kinase domain an attractive site for small-molecule intervention [2,3]. The clinical success of several kinase-fusion-targeted therapies underscores the feasibility of pharmacologically suppressing the catalytic machinery of oncogenic fusion proteins in genetically defined cancers [3,4].

NPM1–TYK2 is an emerging fusion kinase implicated in lymphoid malignancies [5,6]. In this fusion, an NPM1-derived region is linked to the TYK2 kinase-containing region, potentially promoting constitutive activation of the TYK2 catalytic domain and downstream JAK/STAT signaling [5,6]. Because TYK2 is a member of the Janus kinase family and mediates cytokine-associated signaling, aberrant activation of the TYK2 kinase domain may contribute to persistent STAT signaling and tumor-cell proliferation [6,7]. These features suggest that the TYK2 kinase domain within the NPM1–TYK2 fusion may represent a tractable therapeutic target [5,6,8]. Although available TYK2 kinase-domain structures can support ATP-site-directed modeling, target-specific screening knowledge for NPM1–TYK2/TYK2 inhibitor discovery remains limited. In particular, it remains unclear which co-folding/docking–scoring workflow can effectively prioritize compounds with activity in NPM1–TYK2-driven cellular models.

Conventional large-scale structure-based virtual screening is most commonly performed using fixed-receptor docking. In such workflows, a predefined protein conformation is used as the receptor, and virtual screening mainly relies on extensive sampling and scoring of ligand binding poses. Although this strategy has been widely used in drug discovery, its ability to account for receptor flexibility, ligand-induced conformational adaptation, and alternative binding-site conformations is inherently limited [9,10,11]. This limitation is particularly relevant for kinase targets, whose ATP-binding pockets can undergo local conformational changes that influence ligand recognition and scoring.

Recent advances in protein–ligand co-folding models provide a new opportunity to address this limitation. Methods such as AlphaFold 3 (AF3) and Protenix can generate protein–ligand complex conformations directly, thereby allowing ligand placement and local receptor conformational adaptation to be considered within the same modeling process [12,13]. However, plausible complex generation does not necessarily guarantee reliable virtual-screening performance, and confidence metrics from structure-prediction models may not directly translate into effective enrichment of experimentally active compounds [14,15]. Therefore, the practical value of co-folding-based virtual-screening workflows should be evaluated empirically using target-specific experimental activity data. Recent systematic benchmark studies, including Shen et al., have demonstrated the promise of AlphaFold3-like approaches for retrospective virtual screening and pose generation, while also highlighting limitations in computational cost and generalizability. The remaining practical question is whether co-folding-derived complex modeling can be operationalized for commercial-library-scale prospective screening and experimental hit discovery [14,15].

Virtual screening performance is influenced by multiple factors, including receptor model selection, ligand pose generation, docking protocol, scoring function, and rescoring strategy [9,10,16,17]. A workflow that performs well for one target or dataset may not provide robust early enrichment for another [14,15]. This issue is particularly relevant for rare or emerging fusion kinase targets such as NPM1–TYK2, where limited target-specific chemical matter and the absence of an experimentally evaluated screening workflow increase the risk of false-positive prioritization in prospective screening.

In this study, we developed and prospectively applied a co-folding-rescoring workflow for NPM1-TYK2/TYK2 inhibitor discovery. Thirteen pose-generation and scoring pipelines (Table 1) were constructed by combining the co-folding models AlphaFold 3 and Protenix or the docking program Glide and AutoDock Vina with diverse scoring functions or confidence metrics, including RTMScore, Glide SP, AutoDock Vina, pLDDT, ipTM, pTM, and GPDE [12,13,18,19,20,21,22]. Using an in-house Ba/F3-NPM1–TYK2 activity dataset comprising 4608 experimentally annotated compounds, we retrospectively evaluated the early-enrichment performance of these workflows.

Among the evaluated workflows, AF3 + Vina achieved the numerically highest BEDROC value, closely comparable to Protenix + Vina, and was selected for prospective screening of more than 300,000 compounds from the Specs library. This prospective workflow integrated AF3-based protein–ligand complex modeling with the TYK2 kinase domain, Vina rescoring, drug-likeness assessment, physicochemical-property analysis, clustering-based diversity selection, and binding-mode-guided prioritization [12,20]. Experimental testing identified FV13 as a cell-active primary hit, and analog exploration further identified TX14 as an analog with improved TYK2 enzymatic potency. Together, this work demonstrates that co-folding-derived protein–ligand complex conformations, when combined with empirical rescoring and target-specific retrospective evaluation, can be translated into a practical large-scale prospective virtual-screening workflow for NPM1–TYK2/TYK2 inhibitor discovery.

## 2. Results

### 2.1. Overview of the Co-Folding–Scoring Workflow for NPM1–TYK2 Inhibitor Discovery

**Figure 1:**
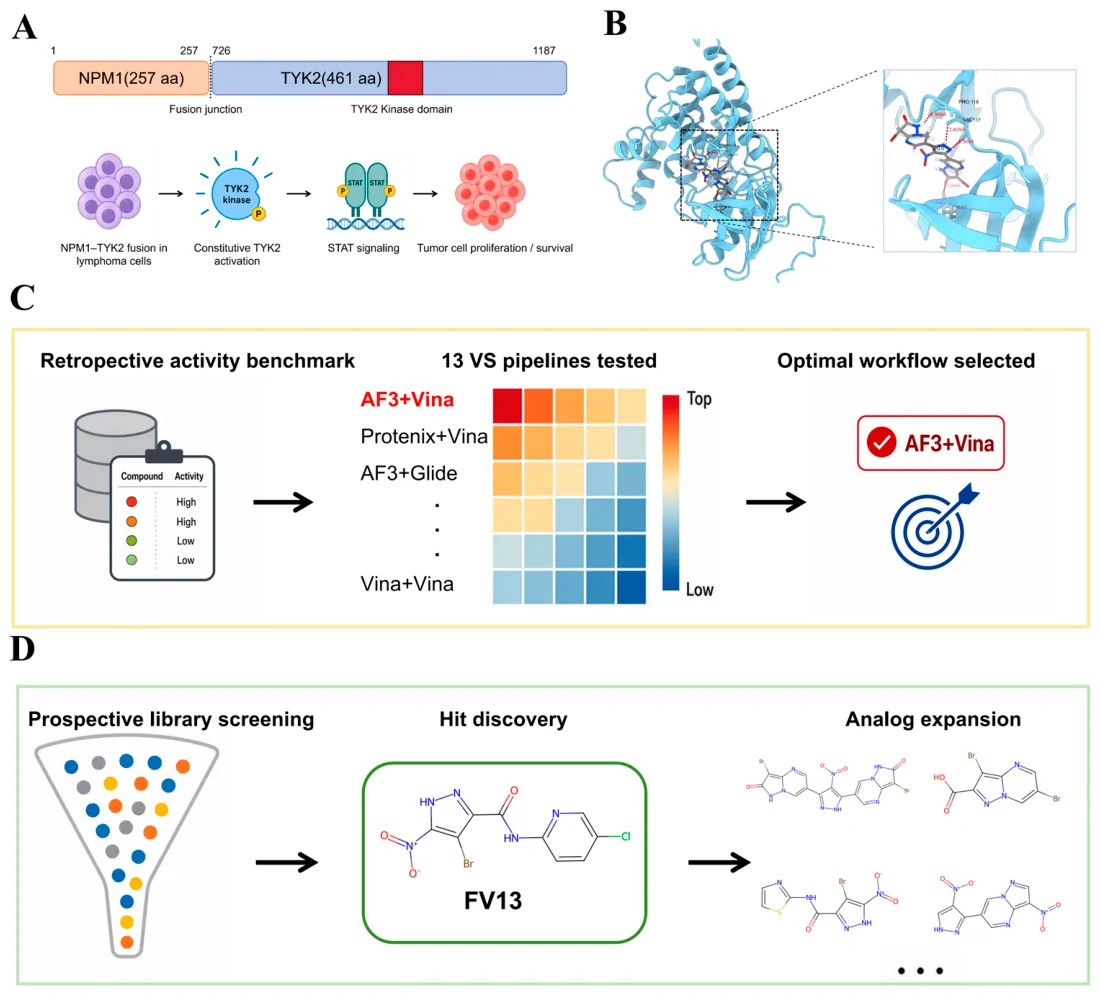
Overview of the co-folding–scoring workflow for NPM1–TYK2 inhibitor discovery. (**A**) Schematic illustration of the NPM1–TYK2 fusion protein and its proposed oncogenic signaling through constitutive TYK2 activation, downstream STAT signaling, and tumor-cell proliferation. (**B**) AF3-derived TYK2 kinase-domain model highlighting the ATP-binding pocket used as the structural context for virtual screening. (**C**) Retrospective workflow evaluation using an internal activity-annotated dataset to compare 13 co-folding/docking–scoring pipelines, leading to the selection of AF3 + Vina. (**D**) Prospective screening and hit-expansion strategy, including Specs library screening, experimental hit discovery of FV13, and analog exploration leading to TX14.

The overall design of this study is summarized in Figure 1. To discover small-molecule inhibitors targeting NPM1–TYK2, we focused on the TYK2 kinase domain and its ATP-binding pocket as the druggable catalytic region of the fusion kinase. NPM1–TYK2 contains an NPM1-derived region fused to a TYK2 kinase-containing region and is proposed to drive constitutive TYK2 activation, downstream STAT signaling, and tumor-cell proliferation (Figure 1A) [5,6]. Because the NPM1–TYK2 fusion retains the TYK2 kinase domain, available TYK2 kinase-domain structures provide a structural basis for ATP-site-directed modeling. We therefore evaluated whether TYK2 kinase-domain-centered co-folding/docking–scoring workflows could enrich NPM1–TYK2-active compounds using target-specific experimental data (Figure 1B) [6,12]. Unlike conventional fixed-receptor docking workflows, the co-folding-based pipelines evaluated here generated protein–ligand complex conformations with the TYK2 kinase domain, thereby allowing receptor and ligand conformations to be modeled jointly before empirical rescoring.

To identify a suitable virtual-screening workflow for NPM1–TYK2/TYK2 inhibitor discovery, we systematically evaluated 13 co-folding/docking–scoring pipelines by combining representative pose-generation methods, including AlphaFold 3, Protenix, AutoDock Vina and Glide, with diverse scoring functions or confidence metrics, including RTMScore, Glide SP, AutoDock Vina, pLDDT, ipTM, pTM, and GPDE (Table 1) [12,13,18,19,20,21,22]. These pipelines were assessed using an in-house Ba/F3-NPM1–TYK2 retrospective activity dataset comprising 4608 experimentally annotated compounds. Among the evaluated workflows, AF3 + Vina ranked first in both EF@3% and BEDROC analyses, although its BEDROC value was nearly identical to that of Protenix + Vina. Based on its overall metric profile, AF3 + Vina was selected for subsequent prospective screening (Figure 1C) [17].

Guided by this retrospective benchmark evaluation, we applied the AF3 + Vina workflow to a prospective screen of the Specs commercial library containing more than 300,000 compounds. In this workflow, AF3 was used for protein–ligand complex modeling, and the resulting complex poses were subsequently rescored using AutoDock Vina [12,20,22]. The screening cascade further integrated drug-likeness filtering, physicochemical property assessment, ring-system analysis, clustering-based diversity selection, and binding-mode-guided manual prioritization [23,24,25,26,27,28,29]. This multi-parameter prioritization strategy yielded 30 compounds for experimental validation. Subsequent wet-lab testing identified FV13 as a cell-active primary hit, and analog exploration of the FV13-related chemotype led to TX14, an analog with improved TYK2 enzymatic potency (Figure 1D). Together, this workflow connects co-folding-based pose generation, retrospective benchmark evaluation, prospective virtual screening, and early analog exploration for NPM1–TYK2/TYK2 inhibitor discovery.

### 2.2. Construction of an In-House Ba/F3-NPM1–TYK2 Retrospective Activity Dataset and Workflow Evaluation

**Figure 2:**
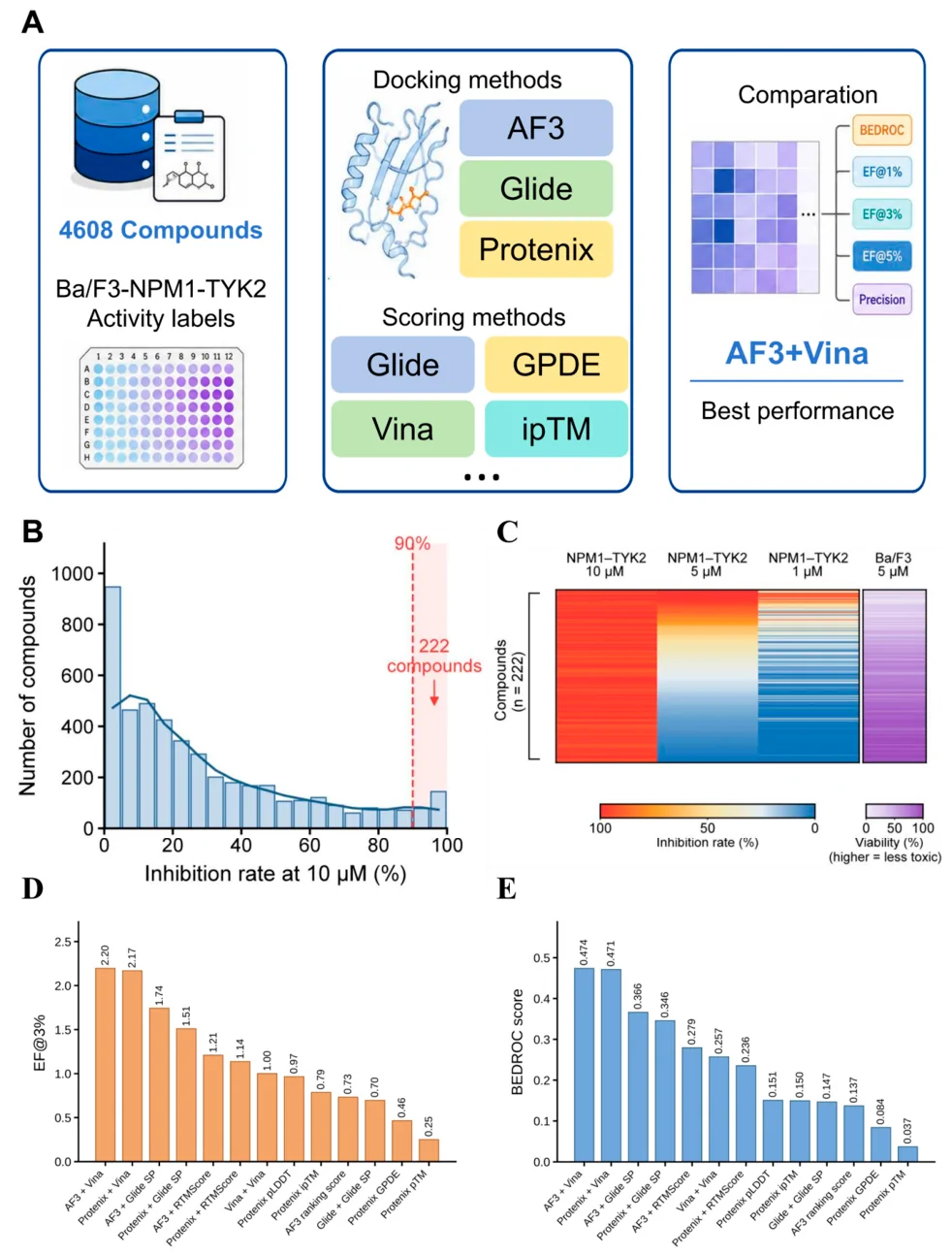
Construction of an in-house Ba/F3-NPM1–TYK2 retrospective activity dataset and retrospective workflow evaluation. (**A**) Schematic overview of the in-house benchmark and virtual screening workflow evaluation strategy. A total of 4608 internal compounds with Ba/F3-NPM1–TYK2 activity labels were used to evaluate co-folding–scoring pipelines. (**B**) Distribution of 10 μM inhibition rates across the 4608-compound benchmark dataset. Compounds with ≥90% inhibition were defined as the high-inhibition subset. (**C**) Lower-concentration activity and parental Ba/F3 counter-screening of the 222-compound high-inhibition subset. Parental Ba/F3 measurements were additionally used for the supplementary benchmarking analysis. (**D**) EF@3% comparison of 13 virtual screening pipelines. (**E**) BEDROC score comparison of the same pipelines.

To establish a target-specific experimental reference for virtual screening workflow evaluation, we constructed an in-house Ba/F3-NPM1–TYK2 retrospective activity dataset. This dataset contained 4608 internal compounds with experimentally annotated cellular activity in Ba/F3-NPM1–TYK2 cells. Each compound was evaluated using a cell-based MTT assay at a uniform screening concentration of 10 μM, and the resulting single-point inhibition rate was used as the primary activity annotation for retrospective workflow evaluation (Figure 2A) [30].

Importantly, this 4608-compound dataset was independent of the prospective Specs library screening campaign. It was not generated from the >300,000-compound Specs screen but instead served as an internally annotated retrospective evaluation set to evaluate whether different computational pipelines could prioritize experimentally active compounds for the NPM1–TYK2 target. Thus, the purpose of this dataset was to provide a target-specific experimental activity reference for early-enrichment assessment rather than to report newly discovered hits.

To assess the chemical-space relationship between the retrospective benchmark set and the prospective Specs screening library, we compared key physicochemical descriptors, Bemis–Murcko scaffold diversity, exact SMILES overlap, and ECFP4 nearest-neighbor similarity (Figure S1A–D) [23,24,25,31]. Overall, the internal benchmark set and the Specs library exhibited broadly similar physicochemical profiles, with particularly close agreement in HBD, TPSA, rotatable bond count, and LogP-related properties (Figure S1C). The internal set also retained substantial structural diversity, comprising 3646 unique Bemis–Murcko scaffolds among 4333 filtered compounds (Figure S1A) [25]. Consistent with this overlap, ECFP4 nearest-neighbor analysis showed that each internal compound had a corresponding nearest neighbor in the Specs library, with a median maximum Tanimoto similarity of 0.531 and a mean value of 0.575 (Figure S1B) [23,24,31]. Moreover, 20.1% of internal compounds had a Specs neighbor with Tanimoto similarity ≥0.70, whereas exact SMILES overlap was restricted to only two compounds (Figure S1D), indicating that the observed relationship was not attributable to duplicated structures. Collectively, these findings support substantial chemical-space overlap between the benchmark set and the prospective screening library, while preserving dataset independence.

The activity distribution of the 4608 compounds at 10 μM covered a broad range of inhibition values, enabling both continuous ranking-based evaluation and threshold-based active-subset definition (Figure 2B). The 10 μM single-point inhibition data were used for retrospective evaluation of the virtual-screening pipelines. For enrichment-based analyses, compounds showing ≥50% inhibition at 10 μM were defined as active compounds. In addition, compounds showing ≥90% inhibition at 10 μM were defined as a high-inhibition subset for subsequent experimental characterization, yielding 222 compounds.

The 222 high-inhibition compounds were subsequently characterized at lower single-point concentrations of 5 μM and 1 μM in Ba/F3-NPM1–TYK2 cells. In parallel, the same compounds were counter-screened in parental Ba/F3 cells at 5 μM to evaluate potential nonspecific cytotoxicity. These lower-concentration and control-cell measurements were used for secondary experimental characterization (Figure 2C). Parental-cell viability was used for the supplementary enrichment analysis reported below and in Table S1.

Using this experimentally annotated benchmark, we compared 13 virtual screening pipelines that integrated different docking/modeling and scoring strategies [12,13,18,19,20,21,22]. The goal of this retrospective evaluation was to identify a workflow withstrong overall early-enrichment capability for NPM1–TYK2-active compounds. BEDROC was used as the primary workflow-selection metric because it emphasizes early retrieval of cell-active compounds [9,10,16,17].

Among the 13 workflows, AF3 + Vina achieved the numerically highest BEDROC (0.474), which was nearly identical to Protenix + Vina (0.471) and higher than conventional Vina + Vina (0.257) (Figure 2E; Table S1). AF3 + Vina also ranked first in the EF@3% analysis (Figure 2D) [17]. These results indicated that AF3 + Vina and Protenix + Vina showed comparable top-tier early enrichment in this dataset; AF3 + Vina was taken forward for prospective screening based on its numerically highest BEDROC and first-ranked EF@3% [12,20].

We also used parental Ba/F3 viability at 5 μM to distinguish activity-positive compounds from those showing marked parental-cell toxicity. Among the 222 high-inhibition compounds, 52 had viability below the operational 40% cutoff and were excluded from the positive hit set, leaving 170 compounds that met both the activity and viability criteria. The evaluation cohort retained all 222 compounds, with 170 positive and 52 negative labels. AF3 + Vina ranked first among all 13 workflows, with BEDROC 0.918, followed by Protenix pLDDT (0.913; Table S1). This counter-screen complements the cellular benchmark but does not establish direct TYK2 inhibition.

Together, this in-house benchmark dataset provided a target-specific experimental foundation for virtual screening workflow selection. By integrating broad activity annotation, lower-concentration follow-up testing, control-cell counter-screening, and multi-metric retrospective evaluation, we identified AF3 + Vina as a suitable early-enrichment workflow for NPM1–TYK2 inhibitor discovery.

### 2.3. Prospective Virtual Screening of the Specs Library Using the AF3 + Vina Workflow

Based on the retrospective evaluation results, we applied the selected AF3 + Vina workflow to a prospective virtual screening campaign against the Specs commercial library containing more than 300,000 compounds. This workflow was designed to differ from conventional fixed-receptor docking. Rather than screening ligands against a single predefined receptor conformation, AF3 was used to generate protein–ligand complex conformations with the TYK2 kinase domain for each candidate compound, allowing ligand placement and local receptor conformational adaptation to be modeled jointly [12]. The resulting complex poses were subsequently prepared and rescored using AutoDock Vina, enabling compound ranking based on empirical Vina scores [20,22]. This design translated the retrospectively selected co-folding–rescoring strategy into a practical large-scale hit-discovery workflow for NPM1–TYK2/TYK2 inhibitors.

**Figure 3:**
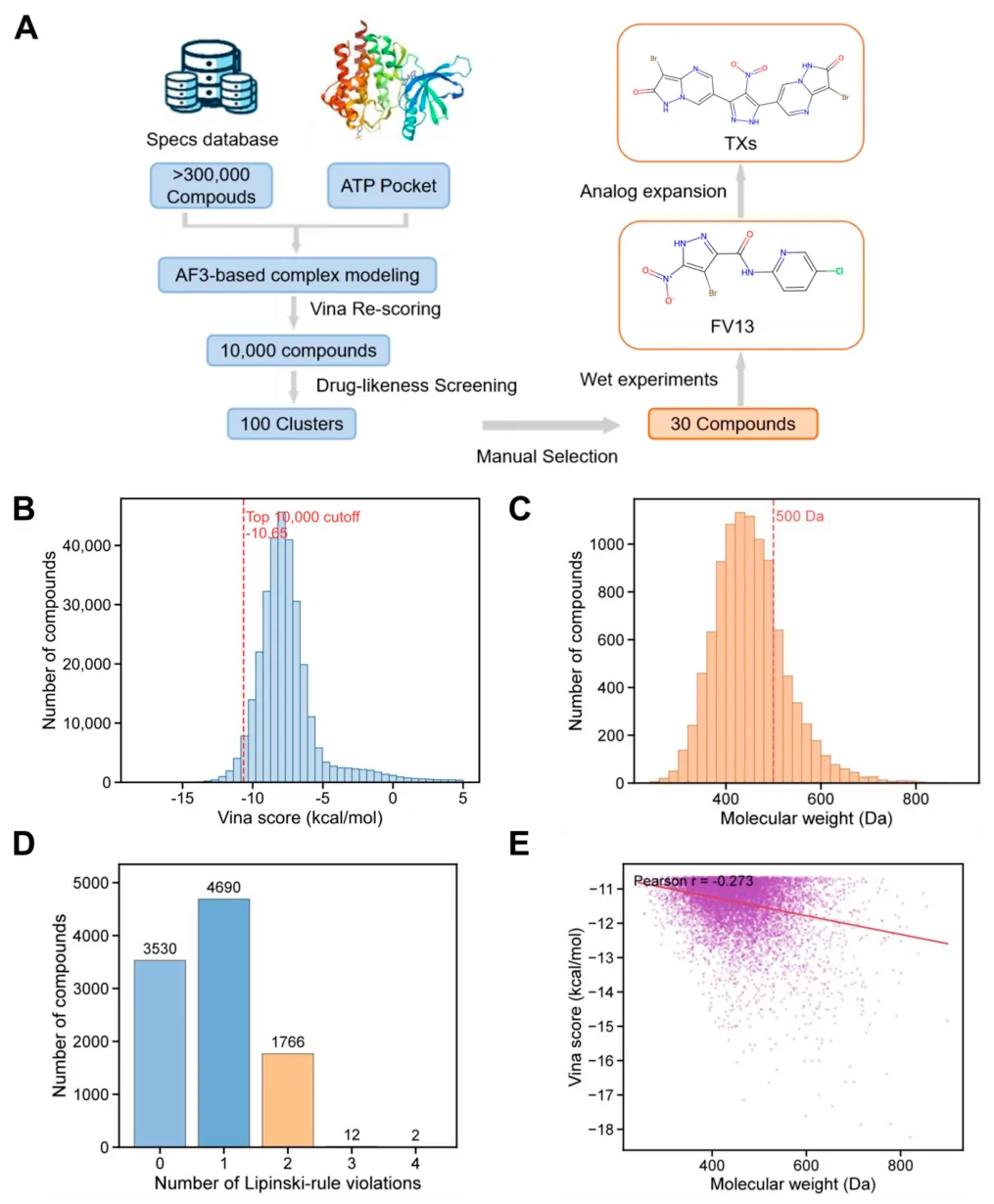
Prospective Specs library screening using the AF3 + Vina workflow. (**A**) Workflow for AF3-based complex modeling, Vina rescoring, filtering, clustering, and final selection of 30 compounds for experimental validation. (**B**) Vina score distribution and top−10,000 cutoff. (**C**) Molecular-weight distribution of the top 10,000 compounds. (**D**) Lipinski-rule violation distribution. (**E**) Molecular weight–Vina score correlation.

The overall prospective screening strategy is summarized in Figure 3A. For each compound from the Specs library, five protein–ligand complex conformations with the TYK2 kinase domain were generated using AlphaFold 3 and only the conformation with the highest ranking score predicted by the AlphaFold 3 confidence model was retained, resulting in approximately 300,000 protein–ligand complex conformations [12]. These retained conformations were then prepared and rescored using AutoDock Vina [20,22]. Based on the rescored Vina scores, the top 10,000 compounds were retained as an enriched candidate pool for subsequent filtering and prioritization. The Vina score distribution of this candidate pool showed enrichment of compounds with favorable predicted binding scores, with the top−10,000 cutoff located at approximately −10.69 kcal/mol and an average Vina score of approximately −11.38 kcal/mol (Figure 3B). These results indicated that the AF3 + Vina workflow prioritized a focused high-scoring subset from the original large-scale commercial library.

Because Vina score alone is insufficient for selecting experimentally testable compounds, we next evaluated the physicochemical and drug-likeness profiles of the top 10,000 compounds [9,10]. Molecular-weight analysis showed that most compounds were distributed within a relatively drug-like molecular size range, although a subset exceeded the Lipinski molecular-weight guideline of 500 Da (Figure 3C) [26]. Consistently, Lipinski-rule violation analysis indicated that most top-ranked compounds had zero to two violations, suggesting that the enriched candidate pool retained a substantial proportion of compounds with acceptable drug-like features (Figure 3D) [26]. Additional physicochemical properties, including LogP, hydrogen-bond donor counts, rotatable bonds, and TPSA, were further examined to provide a broader assessment of developability-related features (Figure S2) [26,32,33]. These analyses supported the need to combine docking-score prioritization with drug-likeness filtering rather than relying solely on predicted binding scores [26,32,34,35].

We also examined whether favorable Vina scores were mainly driven by molecular size or other physicochemical biases. Among the top 10,000 compounds, Vina score showed only a modest correlation with molecular weight, suggesting that molecular size contributed to docking performance but did not fully explain the observed score distribution (Figure 3E). Similarly, additional analyses of LogP and rotatable bond counts showed that individual physicochemical descriptors alone were insufficient to account for the overall docking-score pattern (Figure S2D,F). These observations further supported a multi-parameter prioritization strategy integrating predicted binding score, physicochemical suitability, and medicinal-chemistry considerations [34,35].

**Figure 4:**
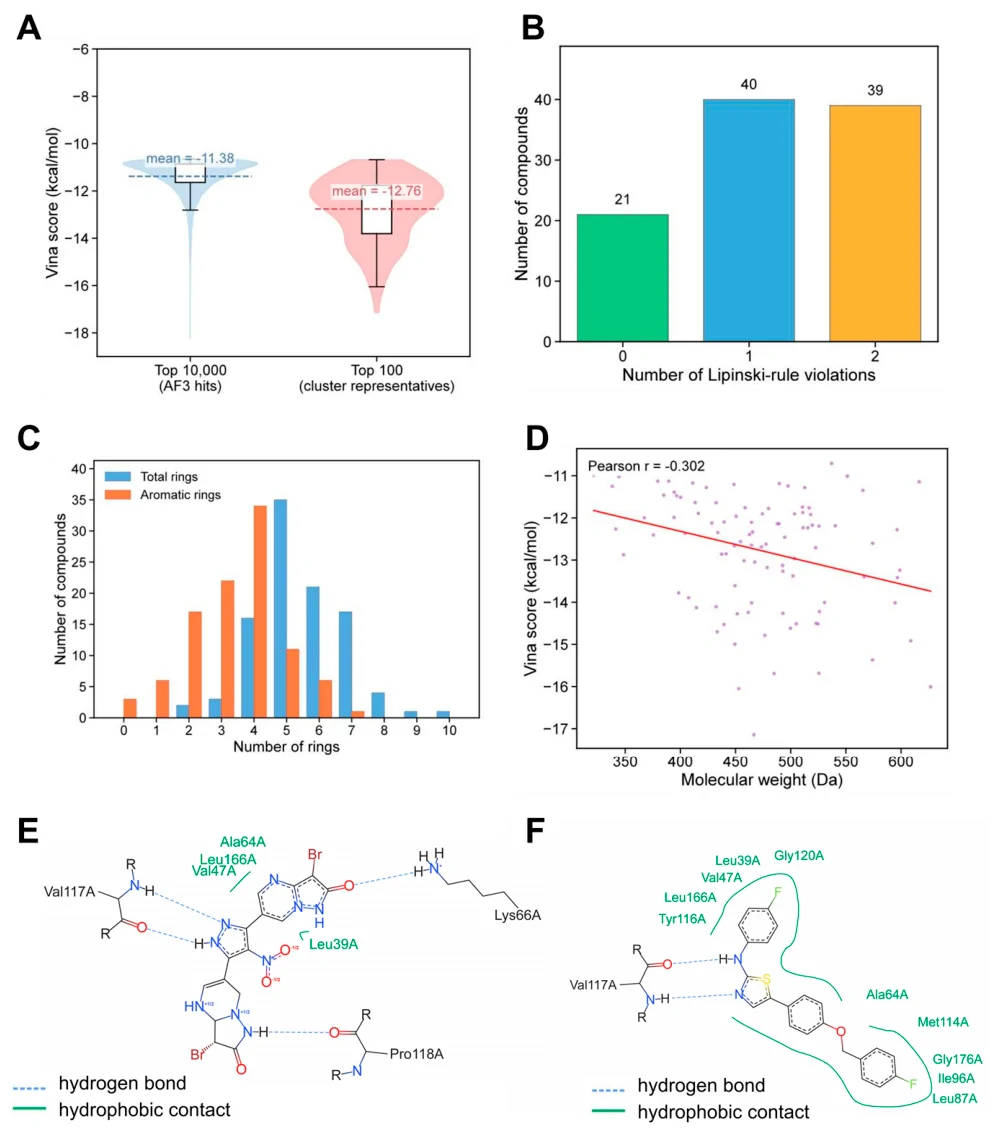
Multi-parameter prioritization of Top 100 cluster representatives. (**A**) Vina score comparison between the top 10,000 compounds and the Top 100 representatives. (**B**) Lipinski-rule violation distribution. (**C**) Ring-system distribution, including total and aromatic ring counts. (**D**) Relationship between molecular weight and Vina score. (**E**,**F**) Representative two-dimensional interaction maps used for binding-mode-guided manual prioritization. Predicted hydrogen bonds and hydrophobic contacts were used to assist the final selection of 30 compounds for experimental validation.

After score-based enrichment and drug-likeness filtering, the candidate compounds were further clustered into 100 clusters based on Morgan fingerprints calculated using RDKit to reduce structural redundancy and preserve scaffold diversity [23,24,27]. From this process, 100 cluster representatives were selected for more detailed evaluation. Compared with the top 10,000 compound pool, the 100 cluster representatives showed a Vina score distribution shifted toward more favorable predicted binding, with an average score of approximately −12.76 kcal/mol compared with approximately −11.38 kcal/mol for the top 10,000 set (Figure 4A). This result suggested that the representative selection process retained compounds with favorable Vina scores while reducing chemical redundancy.

The 100 cluster representatives were then evaluated for drug-likeness and structural diversity [23,25,26,27]. Lipinski-rule violation analysis showed that most representatives contained one or two violations, while a smaller subset had no violations (Figure 4B) [26]. Ring-system analysis revealed a broad distribution of total and aromatic ring counts, indicating that the selected representatives covered diverse ring architectures rather than a single overrepresented chemotype (Figure 4C) [23,25]. In addition, the relationship between molecular weight and Vina score was evaluated within the 100 set to examine potential size-driven scoring bias. The observed correlation suggested that molecular size influenced Vina scoring to some extent, but the selected representatives still covered a range of molecular weights and Vina scores (Figure 4D).

Additional characterization of the 100 representatives, including Vina score distribution, score-category summary, TPSA distribution, molecular-weight comparison, LogP distribution, Lipinski-rule violation distribution, and cluster representation (Figure S3), further supported the selection of the 100 representatives.

After clustering, the 100 representatives were further subjected to structure-based manual prioritization to support the final selection of experimentally testable compounds. This step was not based solely on Vina score. Instead, we visually examined the predicted binding poses within the TYK2 ATP-binding pocket, with particular attention to plausible hydrogen-bonding patterns, hydrophobic contacts, and spatially reasonable occupancy of the ATP-site cleft [28,29]. Representative two-dimensional interaction maps illustrate the type of binding-mode information used during this manual prioritization step (Figure 4E,F). These predicted interaction maps were used as qualitative selection aids rather than as experimental structural validation.

Finally, predicted binding score, drug-likeness, ring-system diversity, scaffold diversity, cluster representativeness, commercial availability, and predicted binding-mode plausibility were integrated to select 30 compounds for wet-lab validation [34,36]. Therefore, the final validation set was not a simple list of the top-scoring compounds but a focused and chemically diverse subset selected through a multi-parameter prioritization process. These 30 compounds were subsequently tested experimentally to identify initial NPM1–TYK2/TYK2-active chemical matter.

### 2.4. Experimental Validation of FV13 and Analog-Based Identification of TX14

**Figure 5:**
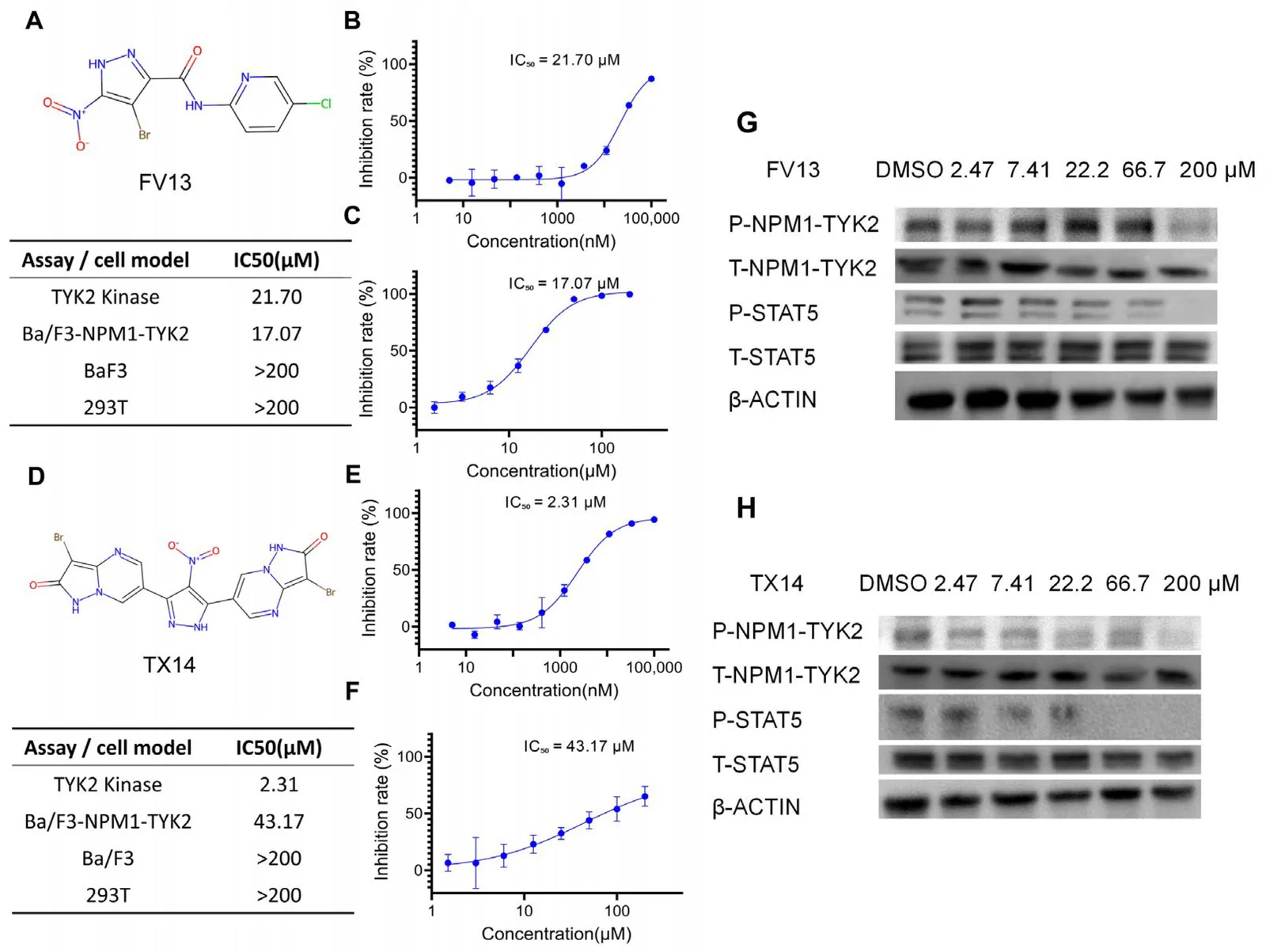
Experimental validation of FV13 and analog-based identification of TX14. (**A**) Chemical structure of FV13 and summary of its IC_50_ values in TYK2 kinase, Ba/F3-NPM1–TYK2, parental Ba/F3, and 293T assays. (**B**) Dose-response curve of FV13 in the TYK2 kinase assay. (**C**) Dose-response curve of FV13 in Ba/F3-NPM1–TYK2 cells. (**D**) Chemical structure of TX14 and summary of its IC_50_ values in TYK2 kinase, Ba/F3-NPM1–TYK2, parental Ba/F3, and 293T assays. (**E**) Dose-response curve of TX14 in the TYK2 kinase assay. (**F**) Dose-response curve of TX14 in Ba/F3-NPM1–TYK2 cells. FV13 was identified as a cell-active primary hit, whereas TX14 showed improved TYK2 kinase potency but weaker cellular activity. Both compounds showed limited cytotoxicity toward parental Ba/F3 and 293T cells. (**G**,**H**) Immunoblot analysis of phosphorylated TYK2, total TYK2, phosphorylated STAT5, and total STAT5 in Ba/F3-NPM1–TYK2 cells treated with FV13 (**G**) or TX14 (**H**) at the indicated concentrations. β-Actin was used as a loading control.

Following multi-parameter prioritization of the prospective screening results, the selected compounds were experimentally evaluated to identify initial NPM1–TYK2/TYK2-active chemical matter. Among the tested candidates, FV13 was identified as a primary hit compound and selected for further characterization. Dose-response analysis showed that FV13 inhibited TYK2 kinase activity with an IC_50_ of 21.70 μM (Figure 5A,B). In Ba/F3-NPM1–TYK2 cells, FV13 also showed concentration-dependent cellular activity, with an IC_50_ of 17.07 μM (Figure 5C). In contrast, FV13 showed limited cytotoxicity against parental Ba/F3 and 293T cells, with IC_50_ values greater than 200 μM, suggesting that its cellular activity was not primarily driven by nonspecific toxicity.

To explore whether the FV13-related chemotype could be further optimized, we evaluated a series of TX analogs. Only a limited subset of TX analogs retained measurable TYK2 kinase activity, indicating that this chemotype had a relatively narrow structural tolerance (Table 2). Among the active analogs, TX14 showed markedly improved biochemical potency against TYK2 kinase. Dose-response analysis demonstrated that TX14 inhibited TYK2 kinase with an IC_50_ of 2.31 μM, representing an approximately 9-fold improvement in enzymatic potency compared with FV13 (Figure 5D,E).

However, the improved biochemical potency of TX14 did not fully translate into improved cellular activity. In Ba/F3-NPM1–TYK2 cells, TX14 showed a cellular IC_50_ of 43.17 μM, which was weaker than that of FV13 (Figure 5F). Similar to FV13, TX14 showed limited cytotoxicity toward parental Ba/F3 and 293T cells, with IC_50_ values greater than 200 μM. These results suggest that TX14 represents a kinase-potency-improved analog, whereas FV13 remains the more cell-active primary hit. The disconnect between biochemical and cellular potency indicates that further optimization of this chemotype should consider not only TYK2 enzymatic inhibition but also cellular exposure, permeability, solubility, and other physicochemical properties [26,32,34,35,37].

To examine whether the cellular activity of FV13 and TX14 was associated with modulation of NPM1–TYK2-related signaling, the phosphorylation levels of TYK2 and STAT5 were examined in Ba/F3-NPM1–TYK2 cells (Figure 5G,H). Both compounds were associated with reduced STAT5 phosphorylation at higher concentrations, with the reduction being most evident at 66.7 and 200 μM. Although neither compound produced a strictly monotonic concentration-dependent change in p-TYK2, their response profiles differed. FV13 showed a clear reduction in p-TYK2 primarily at 200 μM, whereas TX14 maintained lower p-TYK2/total TYK2 levels than the DMSO control across all tested concentrations, with the greatest reduction observed at 200 μM. These observations are consistent with attenuation of NPM1–TYK2-associated signaling, particularly downstream STAT5 phosphorylation, at higher compound concentrations. However, because the p-TYK2 responses were not strictly concentration-dependent, these results should be regarded as supportive pathway-level evidence rather than definitive confirmation of direct and selective cellular TYK2 inhibition.

The biochemical selectivity profiles of FV13 and TX14 were evaluated against JAK1, JAK2, and JAK3 under target-specific assay conditions. FV13 exhibited an IC_50_ of 56.75 μM against JAK1, whereas its IC_50_ values against JAK2 and JAK3 were above 100 μM. Relative to its previously determined TYK2 IC_50_ of 21.70 μM, these results corresponded to an apparent 2.6-fold separation from JAK1 and greater than 4.6-fold separation from JAK2 and JAK3. TX14 inhibited JAK1 with an IC_50_ of 2.51 μM, similar to its TYK2 IC_50_ of 2.31 μM, indicating limited discrimination between TYK2 and JAK1. In contrast, its IC_50_ values against JAK2 and JAK3 were 31.59 and 16.41 μM, corresponding to approximately 13.7- and 7.1-fold apparent selectivity over TYK2, respectively.

As a preliminary counter-screen against a representative non-kinase ATP-dependent enzyme, FV13 and TX14 were evaluated against POLQ (Table 3). FV13 did not reach 50% inhibition within the tested concentration range (IC_50_ > 100 μM), whereas TX14 inhibited POLQ with an IC_50_ of 13.58 μM, corresponding to an approximately 5.9-fold lower potency than that observed against TYK2. These results provide an initial assessment of non-kinase ATP-dependent off-target activity but should not be interpreted as a comprehensive selectivity profile.

Together, these experimental results identified FV13 as an initial cell-active hit from the prospective screening campaign and identified TX14 as an analog with substantially improved TYK2 kinase potency. This provides a chemical starting point for subsequent medicinal-chemistry optimization of NPM1–TYK2/TYK2 inhibitors.

## 3. Discussion

An important challenge in structure-based virtual screening is not simply generating plausible protein–ligand complexes, but determining whether these predictions can improve the prioritization of experimentally active compounds at a practically useful scale. Our study addresses this challenge in the context of NPM1–TYK2, an oncogenic fusion kinase for which target-specific discovery strategies remain relatively underexplored. By systematically comparing 13 co-folding/docking–scoring pipelines against 4608 experimentally annotated compounds and subsequently applying the selected workflow to more than 300,000 commercial molecules, we connect model selection, large-scale virtual screening, and experimental hit identification within a single target-focused framework. This integration represents the main advance of the work: it moves protein–ligand co-folding beyond retrospective structural assessment toward an experimentally informed strategy for prospective small-molecule discovery.

The benchmark reveals a particularly useful complementarity between co-folding-based pose generation and empirical affinity scoring. AF3 + Vina achieved a BEDROC value of 0.474, compared with 0.257 for the conventional fixed-receptor Vina + Vina control under the evaluated conditions. (Figure 2E; Table S1). Importantly, Protenix + Vina also performed strongly (BEDROC = 0.471). Rather than making the outcome dependent on a marginal distinction between two co-folding models, this near-equivalence reinforces the broader methodological insight that co-folding-derived complex poses can become more useful for screening when coupled with an appropriate rescoring strategy. The fixed-receptor Vina comparison provides an informative reference because the scoring function is shared, highlighting the contribution of the upstream structural-modeling workflow to early compound recognition. Together, these observations argue for evaluating pose generation and scoring as a coupled system, rather than assuming that either a more sophisticated structural predictor or a higher structural-confidence score will necessarily yield better virtual-screening rankings.

A further strength is that workflow selection was based on a substantial target-relevant experimental collection rather than a generic docking benchmark alone. The retrospective dataset encompassed a broad range of Ba/F3-NPM1–TYK2 cellular activities and retained extensive scaffold diversity, including 3646 distinct Bemis–Murcko scaffolds among 4333 quality-controlled compounds. We also examined potential confounding from general cytotoxicity. Of the 222 compounds showing at least 90% inhibition in the primary 10 μM assay, 170 satisfied an operational parental Ba/F3 viability criterion, whereas 52 did not. In a supplementary analysis of this counter-screened subset, AF3 + Vina remained the highest-ranked workflow (BEDROC = 0.918), closely followed by Protenix + pLDDT (0.913; Table S1). These complementary analyses make the screening decision more biologically informed and show that the leading workflow remains competitive under an additional toxicity-aware classification. Because the counter-screen covered an activity-enriched subset and used a different test concentration, however, this result is best interpreted as supplementary support rather than a direct measurement of TYK2-specific target engagement.

The prospective experiment demonstrates that the selected workflow can be operationalized beyond benchmark evaluation. For each Specs compound, AF3-generated complex conformations were subjected to Vina rescoring, after which approximately 10,000 top-ranked candidates were considered for physicochemical filtering, structural clustering, and binding-mode-guided review. Reducing this pool to 100 cluster representatives and ultimately 30 experimentally tested compounds enabled the screening effort to balance computational ranking with chemical diversity and practical testability (Figure 3 and Figure 4). Notably, the internal benchmark and Specs library had only two exact SMILES matches, although they occupied partially related chemical space (median maximum ECFP4 nearest-neighbor similarity = 0.531; Figure S1). This limited exact overlap reduces the risk that the apparent relevance of the benchmark to prospective screening is driven mainly by repeated chemical identities. More broadly, this staged screening strategy illustrates how large-scale co-folding outputs can be translated into a focused and experimentally actionable shortlist without relying on docking score alone.

Experimental follow-up demonstrates that this strategy delivered chemically useful starting points. FV13 inhibited TYK2 kinase activity (IC_50_ = 21.70 μM) and Ba/F3-NPM1–TYK2 cell proliferation (IC_50_ = 17.07 μM), while showing IC_50_ values above 200 μM in parental Ba/F3 and 293T cells (Figure 5; Table 2). Exploration of the FV13-related chemical series subsequently identified TX14 with a TYK2 biochemical IC_50_ of 2.31 μM, representing an approximately 9.4-fold potency improvement over FV13. This gain establishes that the initial hit series can accommodate modifications with substantial effects on enzyme-level activity and supplies a concrete structure–activity direction for further optimization. At the same time, TX14 showed weaker Ba/F3-NPM1–TYK2 cellular activity (IC_50_ = 43.17 μM), underscoring that successful biochemical optimization must ultimately be integrated with compound exposure and cellular pharmacology. The complementary profiles of FV13 and TX14 therefore provide two informative optimization starting points: cellular activity for FV13 and biochemical potency for TX14.

Additional biochemical counter-screening and signaling experiments strengthen the characterization of these early hits and clarify the priorities for further development. FV13 displayed an apparent TYK2-versus-JAK1 IC_50_ separation of approximately 2.6-fold and showed IC_50_ values above 100 μM against JAK2, JAK3, and POLQ. TX14, while more potent against TYK2, also inhibited JAK1 at 2.51 μM, indicating that increased kinase potency was accompanied by reduced discrimination within the JAK family (Table 3). In exploratory Western blot experiments, higher concentrations of both compounds were associated with attenuation of downstream STAT5 phosphorylation in Ba/F3-NPM1–TYK2 cells, providing an initial connection between the biochemical findings and a pathway-level cellular readout (Figure 5G,H). Because TYK2 phosphorylation responses were not uniformly concentration-dependent and the signaling measurements were obtained at relatively high concentrations, these results support pathway modulation but do not yet establish selective intracellular TYK2 inhibition. Importantly, the combined enzyme, cellular, selectivity, and signaling readouts establish a more informative pharmacological profile than a docking score or single viability measurement could provide.

The principal significance of this work is therefore both methodological and experimental. It demonstrates how target-specific cellular evidence can guide the selection of a co-folding–rescoring workflow, how this workflow can be deployed across a commercial-scale library, and how prospective hits can initiate meaningful medicinal-chemistry exploration. Further work should assess the robustness of workflow ranking across additional targets and receptor conformations, compare alternative pipelines prospectively, and validate the predicted binding modes experimentally. For FV13 and TX14, matched-concentration counter-screening, replicated short-time-point signaling studies, direct cellular target-engagement assays, and broader kinase selectivity profiling will be valuable, alongside optimization of cellular exposure and potency. These next steps build on the present findings rather than diminish them: the study establishes an experimentally grounded discovery platform and identifies chemical matter from which more potent and selective NPM1–TYK2-directed compounds may be developed.

## 4. Materials and Methods

### 4.1. Materials and Reagents

Compounds FV13 and TX14 were purchased from TargetMol (Shanghai, China) and dissolved in dimethyl sulfoxide (DMSO) to prepare stock solutions of 20 mM and 30 mM, respectively. RPMI-1640 medium (U21-279b, YOBIBIO, Shanghai, China), Dulbecco’s modified Eagle’s medium (DMEM; U21-265B, YOBIBIO), fetal bovine serum (FBS; F8318, Sigma-Aldrich, Burlington, MA, USA), recombinant interleukin-3 (IL-3; 90143ES10, Yeasen, Shanghai, China), and penicillin–streptomycin–glutamine (10378016, Gibco, Grand Island, NY, USA) were used for cell culture. Cell lysis buffer (P0013, Beyotime, Shanghai, China), 10% SDS–PAGE gels (PG113, Epizyme, Cambridge, MA, USA), polyvinylidene difluoride (PVDF) membranes (IPFL00005, Millipore, Burlington, MA, USA), QuickBlock™ Western Blocking Buffer (P0252, Beyotime), and enhanced chemiluminescence (ECL) reagent (P0018S, Beyotime) were used for immunoblotting experiments. Primary antibodies against phospho-TYK2 (Tyr1054/1055; #9321S), total TYK2 (E9H4T; #35615S), phospho-STAT5 (Tyr694; #9351), and total STAT5 (D2O6Y; #94205T) were purchased from Cell Signaling Technology and used at a dilution of 1:1000. The β-actin mouse monoclonal antibody (#3700S, 1:3000, Cell Signaling Technology, Danvers, MA, USA) was used as a loading control. HRP-conjugated anti-mouse IgG (D110087, 1:5000, Sangon Biotech, Shanghai, China) and anti-rabbit IgG (D110058, 1:5000, Sangon Biotech) were used as secondary antibodies.

### 4.2. Construction of the In-House Ba/F3-NPM1–TYK2 Retrospective Activity Dataset

An in-house retrospective activity dataset was constructed using Ba/F3-NPM1–TYK2 cells. A total of 4608 internal compounds were experimentally evaluated by a cell-based MTT assay at a uniform screening concentration of 10 μM. The single-point inhibition rate of each compound was calculated and used as the primary activity annotation for retrospective virtual screening evaluation. For the primary enrichment analysis, compounds showing ≥50% inhibition at 10 μM were defined as active compounds. Compounds showing ≥90% inhibition at 10 μM were defined as the high-inhibition subset. A total of 222 compounds met this criterion and were further evaluated at lower single-point concentrations of 5 μM and 1 μM in Ba/F3-NPM1–TYK2 cells. In parallel, these 222 compounds were counter-screened in parental Ba/F3 cells at 5 μM to assess potential nonspecific cytotoxicity. The parental Ba/F3 measurements were additionally used for the supplementary enrichment analysis described in Section 4.5.

### 4.3. Chemical-Space Comparison Between the Benchmark Set and the Specs Library

To evaluate the chemical-space relationship between the retrospective benchmark set and the prospective Specs screening library, a quality-controlled subset of 4333 internal compounds was compared with the full Specs library. Key physicochemical descriptors, including molecular weight, LogP, hydrogen-bond acceptor count, hydrogen-bond donor count, TPSA, and rotatable bond count, were calculated using RDKit 2023.09.5. Bemis–Murcko scaffolds were extracted to assess scaffold diversity.

To examine structural similarity between the two datasets, ECFP4/Morgan fingerprints were generated, and the maximum Tanimoto similarity from each internal compound to its nearest neighbor in the Specs library was calculated. Exact canonical SMILES overlap between the internal benchmark set and the Specs library was also examined to assess potential duplicate molecules.

### 4.4. Construction of Co-Folding/Docking–Scoring Pipelines

Thirteen co-folding/docking–scoring pipelines were constructed to evaluate the compatibility between different pose-generation methods and scoring or confidence-based ranking strategies. The pose-generation methods included AlphaFold 3, Protenix (v0.4.6; pretrained model v0.2.0), Glide v9.1, and AutoDock Vina v1.2.7, whereas the scoring or ranking methods included AutoDock Vina, Glide SP Glide SP, Schrödinger Suite 2021-2, Build 117, RTMScore model 1, GPDE, pLDDT, pTM, and ipTM.

AlphaFold 3 and Protenix used the same crystallographic TYK2 kinase-domain construct to generate protein–ligand complexes; Glide docking used the prepared kinase-domain structure. Predicted complexes were first converted from CIF to PDB with Open Babel v3.1.1 and processed with Schrödinger Suite 2021-2, Build 117. Preprocessing included bond-order assignment and correction where needed, addition of missing hydrogen atoms, optimization of protonation states and the hydrogen-bond network, and restrained energy minimization to relieve local strain while preserving the overall structure. The prepared complexes were then separated into receptor and ligand components. MGLTools v1.5.7 assigned Gasteiger partial charges and AutoDock atom types, and generated PDBQT inputs for Vina rescoring. For retrospective scoring, each compound was represented by its best-scoring available pose.

For structural assessment, we aligned the AF3 and Protenix receptors to the crystallographic TYK2 kinase domain and calculated global and binding-pocket Cα RMSDs. The same analysis was applied to the receptor models associated with FV13 and TX14. Receptor alignment and RMSD calculation are described in Supplementary Methods S1. For the conventional fixed-receptor Vina control (Vina + Vina), ligands were docked against the prepared crystallographic TYK2 kinase-domain receptor and ranked using Vina scores. Detailed ligand-preparation procedures and docking parameters are provided in Supplementary Methods S2.

### 4.5. Retrospective Virtual Screening Benchmark Evaluation

The primary benchmark used Ba/F3-NPM1–TYK2 inhibition at 10 μM, with ≥50% inhibition defining an active compound. Each pipeline was evaluated on compounds with an available score and activity label; compounds without a score for that pipeline were omitted. Score selection and ranking details are given in Supplementary Methods S3.

BEDROC (α = 80.5) was the primary workflow-selection metric because it emphasizes early recovery of active compounds across the ranked list. EF@1%, EF@3%, and EF@5% were reported as complementary cutoff-based enrichment measures. Pearson r was included as a descriptive measure of score–activity correlation and was not used to select the screening workflow. Metric definitions followed previous studies [16,17].

For the supplementary parental-cell analysis, viability below 40% in parental Ba/F3 cells at 5 μM was used as an operational cytotoxicity criterion. Compounds passing this viability filter and the original activity criterion were labeled positive; compounds failing the viability filter were excluded from the positive hit set and labeled negative. The full 222-compound counter-screened set, including both label classes, was retained for BEDROC evaluation.

### 4.6. Prospective Virtual Screening of the Specs Library

The Specs commercial library containing more than 300,000 compounds was used for prospective virtual screening. For each compound, five protein–ligand complex conformations with the TYK2 kinase domain were modelled by AlphaFold 3. The conformation with the highest-ranking score predicted by the AlphaFold 3 confidence model was retained for each compound, resulting in approximately 300,000 protein–ligand complex conformations.

The retained complex conformations were structure-prepared and rescored using AutoDock Vina. Compounds were ranked according to the rescored Vina scores, and the top 10,000 compounds were retained as an enriched candidate pool for subsequent physicochemical assessment, drug-likeness evaluation, clustering, and manual prioritization.

### 4.7. Physicochemical Assessment, Clustering, and Manual Prioritization

The top 10,000 compounds were evaluated for physicochemical and drug-likeness properties, including molecular weight, LogP, TPSA, hydrogen-bond donor count, hydrogen-bond acceptor count, rotatable bond count, and Lipinski-rule violations. Ring-system properties, including total ring count and aromatic ring count, were also analyzed.

After score-based enrichment and physicochemical assessment, candidate compounds were clustered into 100 structural clusters based on Morgan fingerprints calculated using RDKit. One representative compound was selected from each cluster to reduce structural redundancy and preserve scaffold diversity. The 100 cluster representatives were further evaluated based on Vina score, drug-likeness, ring-system features, molecular diversity, and cluster representativeness.

For final manual prioritization, predicted protein–ligand binding poses were visually inspected within the TYK2 ATP-binding pocket. Particular attention was paid to plausible hydrogen-bonding patterns, hydrophobic contacts, and spatially reasonable occupancy of the ATP-site cleft. The predicted two-dimensional interaction maps were used as qualitative selection aids rather than experimental structural validation. Finally, 30 compounds were selected for experimental validation by integrating predicted Vina score, physicochemical properties, scaffold diversity, cluster representation, commercial availability, and binding-mode plausibility.

### 4.8. TYK2 Kinase Activity Assay

The biochemical inhibitory activity of selected compounds against TYK2 kinase was evaluated using the LANCE Ultra assay. Compounds were dissolved in 100% DMSO and diluted to the indicated concentrations. Compound solutions were transferred into 384-well assay plates using an Echo 650 acoustic liquid handler.

TYK2 kinase was prepared in kinase reaction buffer containing 50 mM HEPES, pH 7.5, 10 mM MgCl_2_, 0.01% Triton X-100, 0.01% BSA, and 2 mM DTT. The kinase solution was added to the assay plate and incubated at room temperature for 10 min. The kinase reaction was initiated by adding substrate solution containing ULight-JAK-1 (Tyr1023) peptide and ATP. The final assay conditions were 5 nM TYK2, 15 nM ULight-JAK-1 (Tyr1023) peptide, and 10 μM ATP. After incubation at room temperature for 30 min, the reaction was terminated by adding detection buffer containing Europium-anti-phosphotyrosine antibody. After incubation at room temperature for 60 min, the LANCE signal ratio at 665/615 nm was measured using an EnVision 2104 Multilabel Reader.

The inhibition rate was calculated using the following equation:

$$Inhibition\;rate\;(\%)=\left[1-\frac{R_{sample}-R_{no-enzyme}}{R_{DMSO}-R_{no-enzyme}}\right]\times 100$$

where *R* represents the LANCE signal ratio at 665/615 nm, *R*_sample_ represents compound-treated wells, *R*_DMSO_ represents DMSO-treated control wells, and *R*_no-enzyme_ represents no-enzyme control wells. Concentration–response curves were fitted using a four-parameter logistic model.

### 4.9. Cell Viability Assay

Ba/F3-NPM1–TYK2 cells, parental Ba/F3 cells, and 293T cells were used to evaluate cellular activity and cytotoxicity. Cells were harvested and seeded into 96-well plates at a density of 10,000 cells per well. After overnight incubation at 37 °C in a humidified incubator with 5% CO_2_, cells were treated with serial dilutions of test compounds or DMSO control for three days.

Cell viability was measured using the MTT assay. Briefly, 10 μL of 5 mg/mL thiazolyl blue tetrazolium bromide (MTT) solution was added to each well and incubated for 4 h. The converted formazan dye was then dissolved by adding MTT solubilization buffer to each well. Absorbance was measured at 570 nm with 650 nm as the reference wavelength. The inhibition rate of each compound was calculated using the following equation:

$$Inhibition\;rate\;(\%)=\left[1-\frac{A_{sample}-A_{blank}}{A_{DMSO}-A_{blank}}\right]\times 100$$

where *A*_sample_ represents the absorbance of compound-treated wells, *A*_DMSO_ represents the absorbance of DMSO-treated control wells, and *A*_blank_ represents the absorbance of blank wells. Concentration–response curves were fitted using a four-parameter logistic model.

### 4.10. JAK-Family Selectivity and POLQ Counter-Screening Assays

The biochemical activities of FV13 and TX14 against JAK1, JAK2, JAK3, and POLQ were determined by Shanghai ChemPartner Co., Ltd. (Shanghai, China) using target-specific biochemical enzyme assays. JAK1, JAK2, and JAK3 were included to evaluate selectivity within the JAK kinase family, whereas POLQ was included as a representative non-kinase ATP-dependent enzyme for preliminary off-target assessment. Compounds were tested using a 10-point, four-fold serial dilution with a highest concentration of 100 μM. The assays were conducted using target-specific ATP concentrations corresponding to the Km,ATP values defined by the service provider.

### 4.11. Western Blot Analysis

Ba/F3-NPM1–TYK2 cells were seeded into six-well plates at a density of 5 × 10^5^ cells per well and treated with FV13 or TX14 at the indicated concentrations (2.47, 7.41, 22.2, 66.7, and 200 μM) for 12 h. DMSO-treated cells served as the vehicle control. Following treatment, cells were harvested and lysed using cell lysis buffer. Protein samples were separated by 10% SDS–PAGE and transferred onto polyvinylidene difluoride (PVDF) membranes. After blocking with QuickBlock™ Western Blocking Buffer, the membranes were incubated overnight with primary antibodies against phosphorylated TYK2, total TYK2, phosphorylated STAT5, total STAT5, and β-actin. After washing, the membranes were incubated with appropriate horseradish peroxidase (HRP)-conjugated secondary antibodies. Protein bands were visualized using enhanced chemiluminescence (ECL) reagents.

### 4.12. Data Analysis and Visualization

Computational data were processed using Python 3.9.16 and RDKit 2023.09.5. Molecular descriptors, fingerprint-based similarity analyses, Bemis–Murcko scaffold analysis, clustering, and statistical summaries were performed using RDKit, NumPy v1.23.5, pandas v1.5.3, and SciPy v1.10.0. For biochemical kinase assays and cell viability assays, concentration–response curves were fitted using a four-parameter logistic model. IC_50_ values were obtained from the fitted curves. Unless otherwise specified, data are presented as mean ± SD.

## 5. Conclusions

In summary, we developed and prospectively applied an experimentally guided, target-specific co-folding–rescoring virtual-screening workflow for NPM1–TYK2/TYK2 inhibitor discovery. Systematic evaluation of 13 pipelines using an in-house dataset of 4608 experimentally annotated compounds identified AF3 + Vina as a leading workflow, outperforming conventional Vina + Vina while achieving performance comparable to Protenix + Vina. By integrating retrospective workflow evaluation, prospective Specs library screening, multi-parameter prioritization, and experimental testing, we identified FV13 as a cell-active primary hit (TYK2 IC_50_ = 21.70 μM; Ba/F3-NPM1–TYK2 cellular IC_50_ = 17.07 μM) and TX14 as an analog with an approximately 9.4-fold improvement in biochemical TYK2 inhibitory potency (IC_50_ = 2.31 μM). Additional biochemical selectivity profiling and Western blot analyses further characterized the pharmacological properties of these compounds and provided preliminary evidence of downstream STAT5 signaling modulation at higher concentrations. Although these compounds remain early-stage micromolar hits, they provide useful starting points and structure–activity information for further optimization, particularly with respect to cellular activity and kinase selectivity. Overall, this study demonstrates that co-folding-based complex modeling can be coupled with empirical rescoring and target-specific activity data to support large-scale prospective virtual screening, providing a practical alternative to conventional fixed-receptor docking workflows for early hit discovery.

## Figures and Tables

**Table 1:** Summary of the 13 co-folding/docking–scoring pipelines evaluated in this study.

Pipeline ID	Docking Method	Scoring Method
P1	AlphaFold 3	AutoDock Vina
P2	AlphaFold 3	Glide
P3	AlphaFold 3	RTMScore
P4	AlphaFold 3	ipTM
P5	Glide	Glide
P6	Protenix	AutoDock Vina
P7	Protenix	Glide
P8	Protenix	RTMScore
P9	Protenix	GPDE
P10	Protenix	ipTM
P11	Protenix	pLDDT
P12	Protenix	pTM
P13	AutoDock Vina	AutoDock Vina

**Table 2:** Biochemical and cellular activities of FV13 and representative active TX analogs.

Compound	Structure	TYK2 Kinase IC_50_ (μM)	Ba/F3-NPM1–TYK2 IC_50_ (μM)
FV13	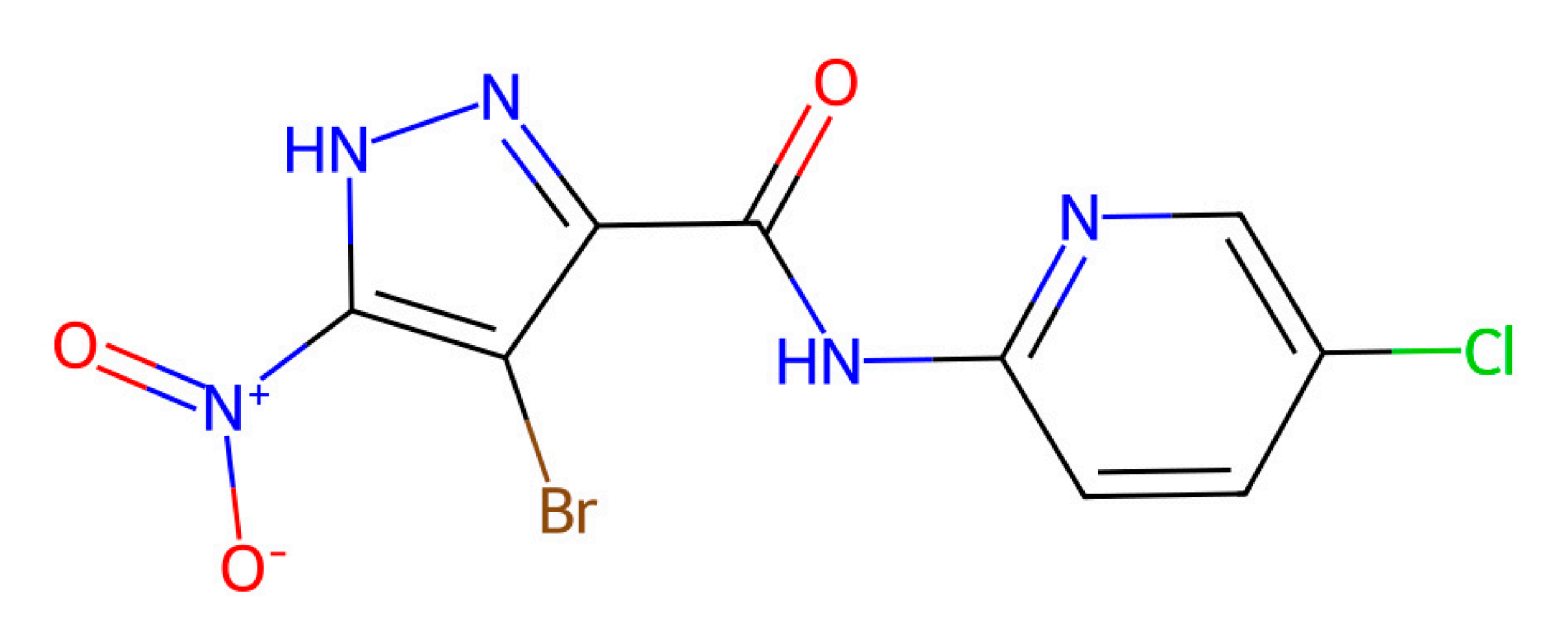	21.70	17.07
TX1	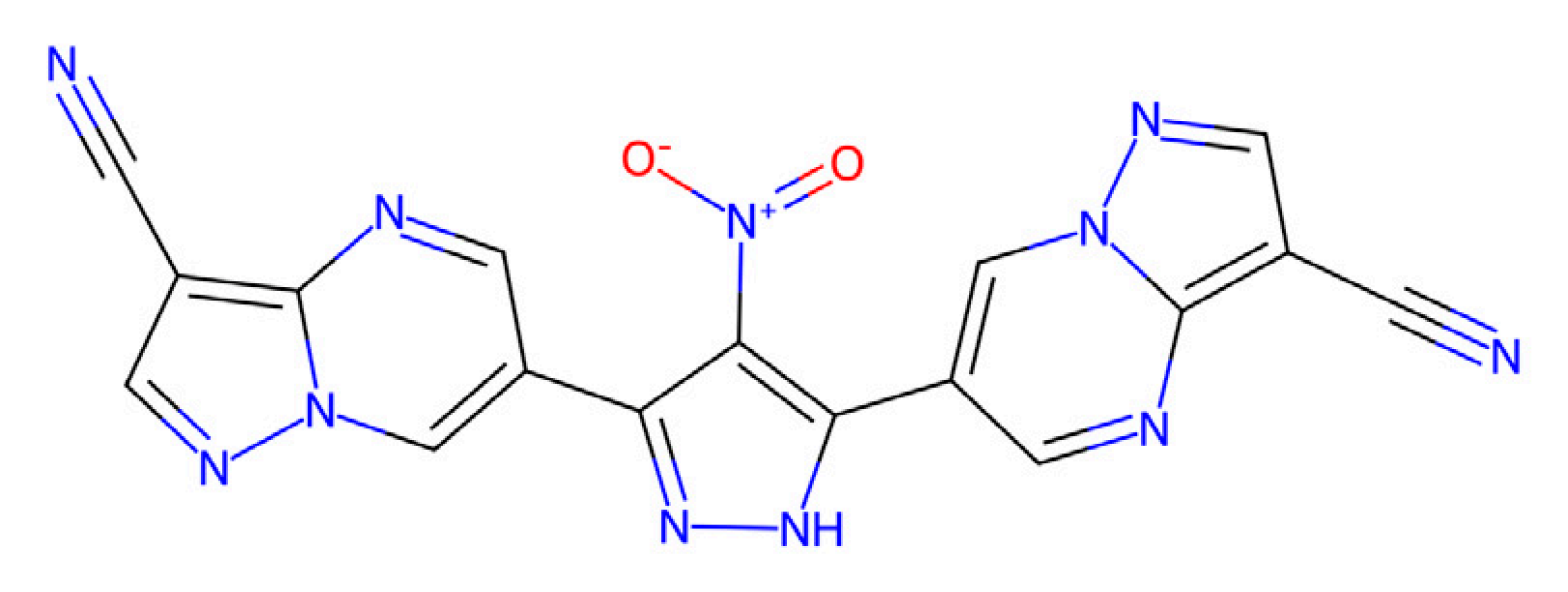	57.25	36.21
TX4	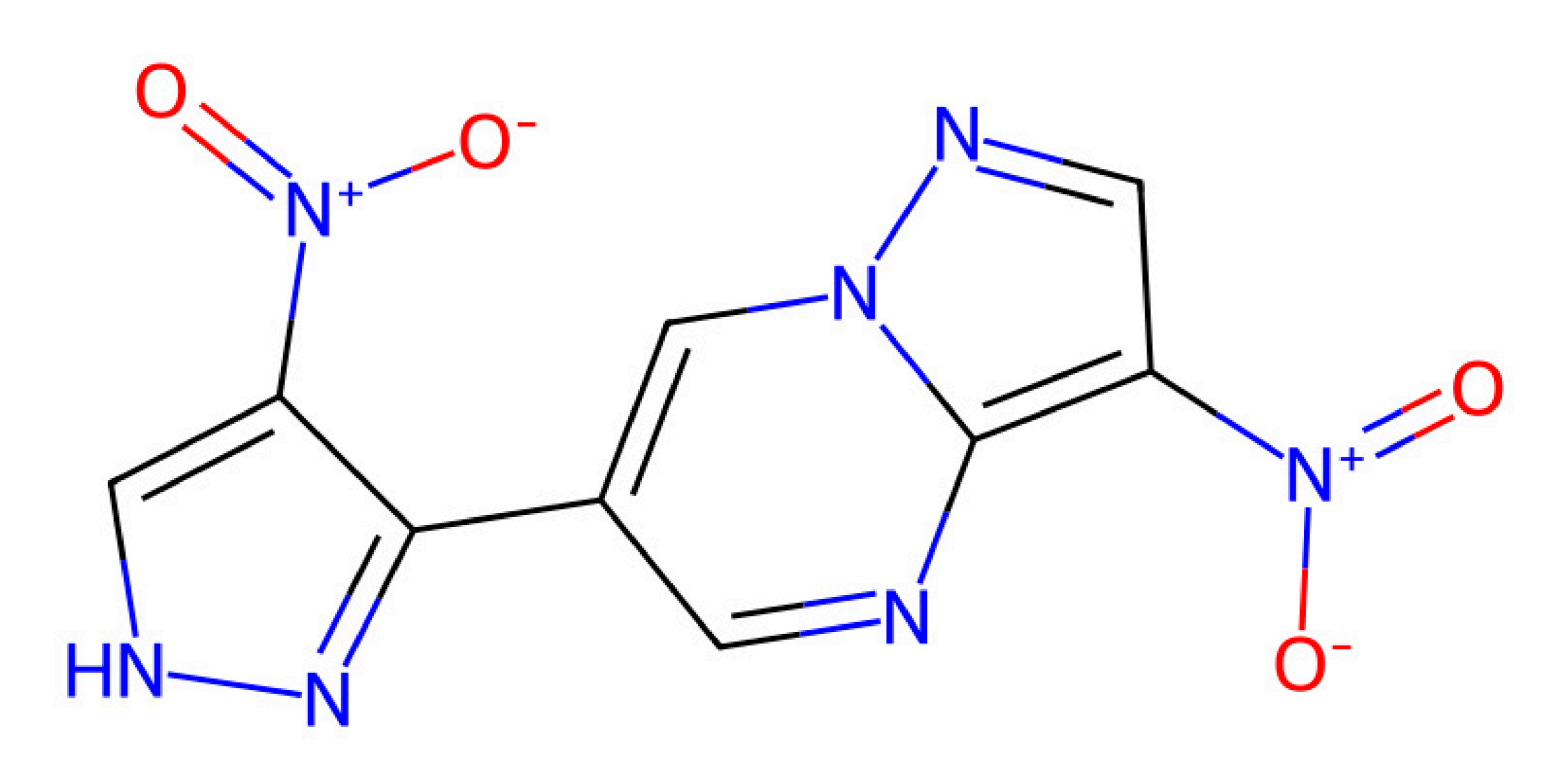	56.78	37.94
TX14	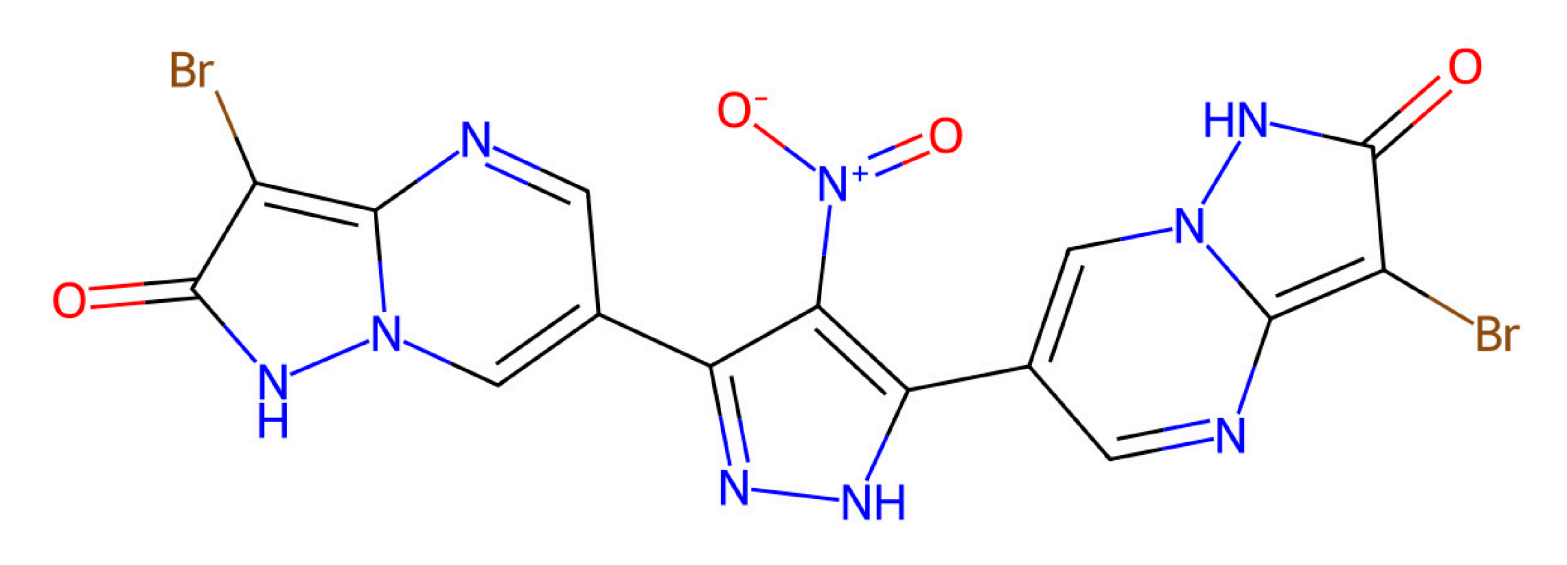	2.31	43.17
TX19	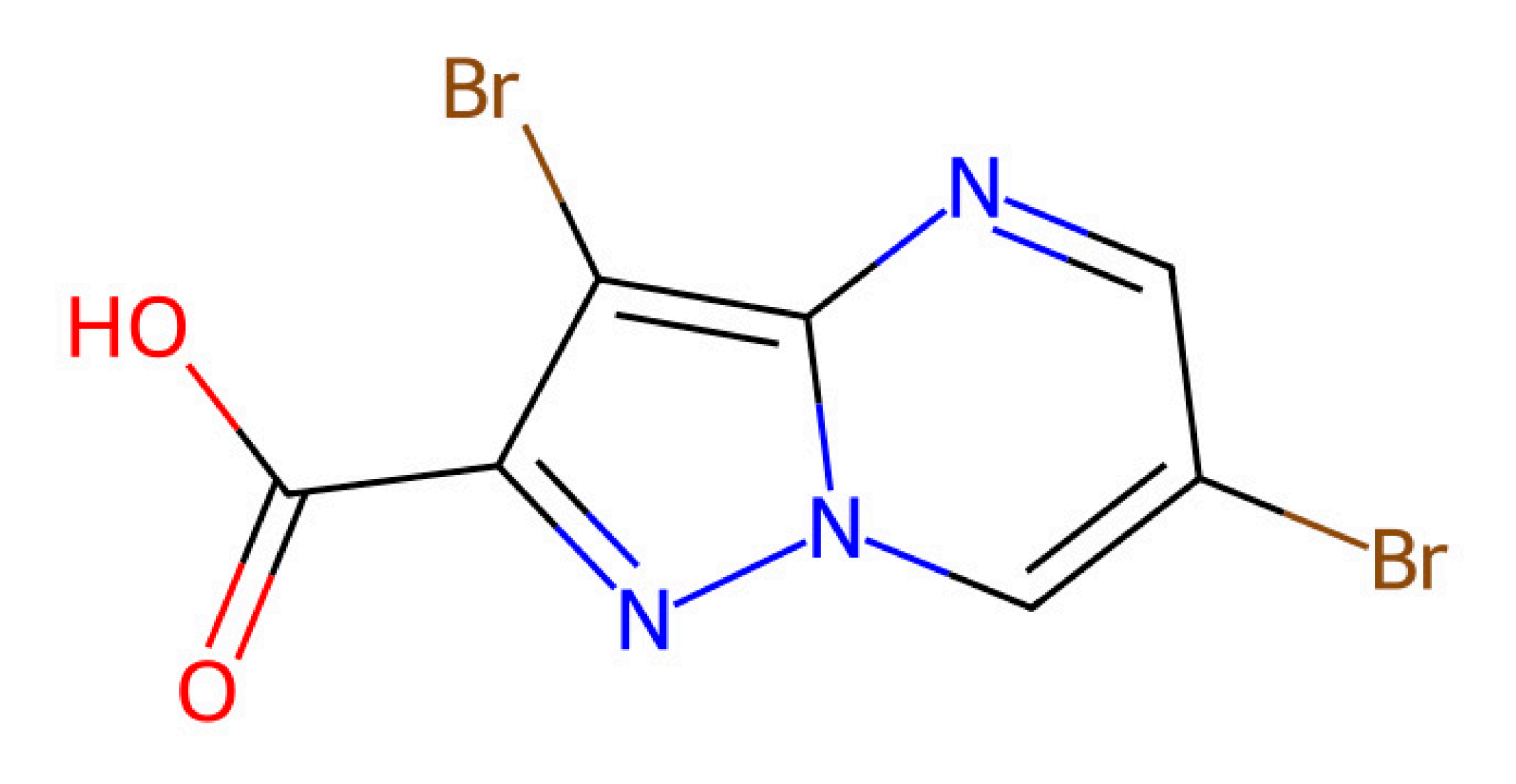	11.04	59.81
TX22	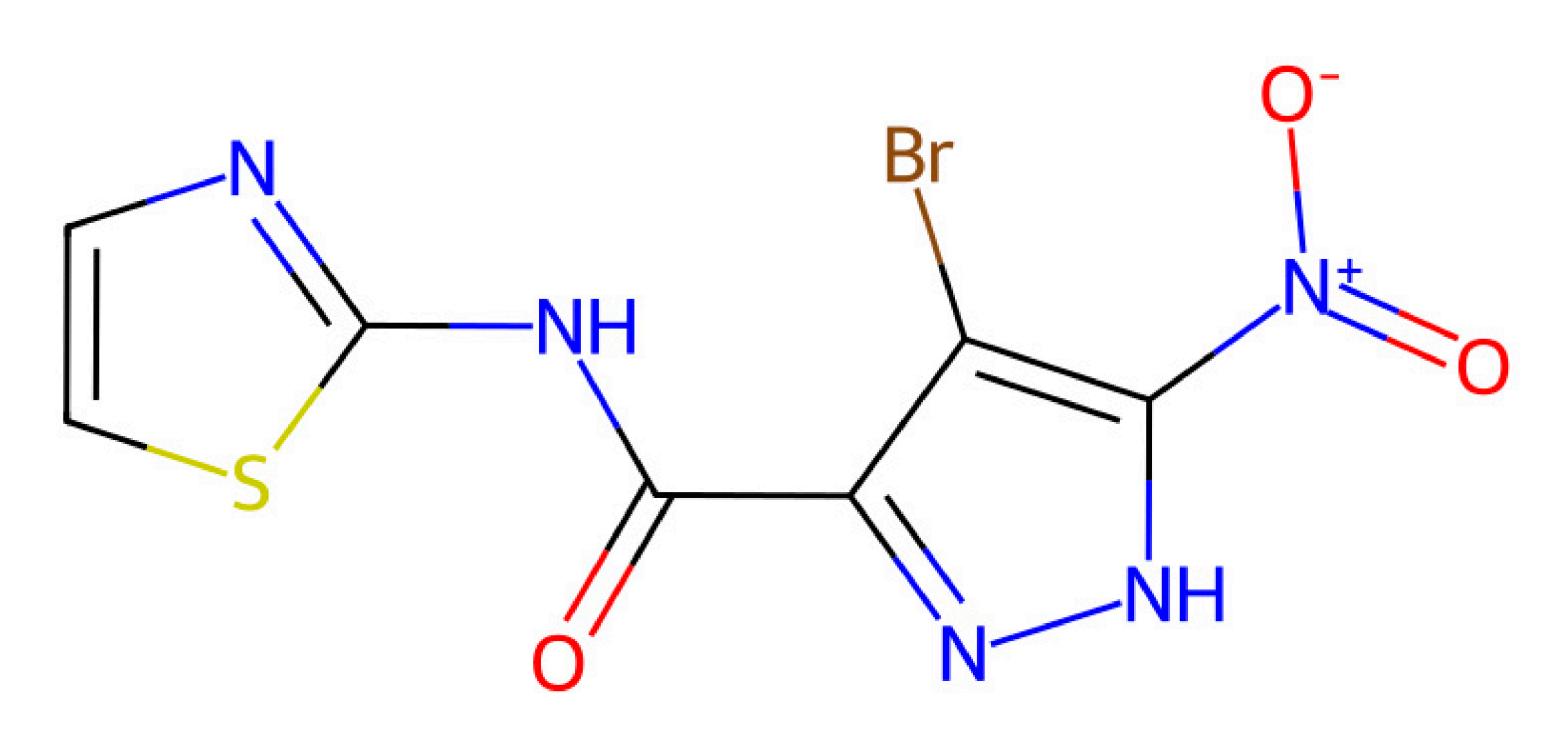	28.70	40.69

**Table 3:** Biochemical activity profiles of FV13 and TX14 against TYK2, JAK-family kinases, and POLQ.

Target	FV13 IC_50_ (μM)	TX14 IC_50_ (μM)
TYK2	21.70	2.31
JAK1	56.75	2.51
JAK2	>100	31.59
JAK3	>100	16.41
POLQ	>100	13.58

## Data Availability

Machine-readable data supporting the findings of this study are publicly available in Zenodo at https://doi.org/10.5281/zenodo.22294606. The deposited datasets include molecular structures and 10 μM Ba/F3-NPM1–TYK2 activity data for the retrospective benchmark set; follow-up activity and parental Ba/F3 counter-screening data for the high-inhibition subset; retrospective benchmarking results for co-folding/docking–scoring pipelines, including enrichment and ranking metrics across different activity thresholds and ranking fractions, together with processed summary metrics used for comparative analysis; compound-level prospective AF3 + Vina screening results for the Specs library, including compound identifiers, molecular structures, physicochemical properties, and Vina rescoring scores; and molecular structures and experimental activity data for the prospectively tested compounds and subsequent TX analogs. Detailed descriptions of the individual datasets and data fields are provided in the README file accompanying the repository. Protein–ligand complex modeling, molecular docking, rescoring, and computational analyses were performed using existing publicly available or commercial software, including AlphaFold 3, Protenix, Glide/Glide SP, AutoDock Vina, RTMScore, Python, RDKit, NumPy, pandas, and SciPy (Python and RDKit versions are specified in Section 4.12). The pLDDT, ipTM, pTM, and GPDE metrics were used as applicable confidence or ranking metrics. No new software package or standalone computational tool was developed as part of this study. Additional methodological details and computational settings are described in the Section 4.

## Supplementary Materials

The following supporting information can be downloaded at: https://www.mdpi.com/article/10.3390/ijms27219864/s1.
