# Supplementary material for: Large-Scale Prospective Virtual Screening Using a Co-Folding-Rescoring Workflow for NPM1-TYK2 Inhibitor Discovery
Source: Int J Mol Sci. 2026 Nov 3;27(21):9864. doi: 10.3390/ijms27219864 (PMC13764033; doi:10.3390/ijms27219864)
Supplement: Supplementary file 1 [file ijms-27-09864-s001.zip › ijms-4626817-supplementary.pdf]

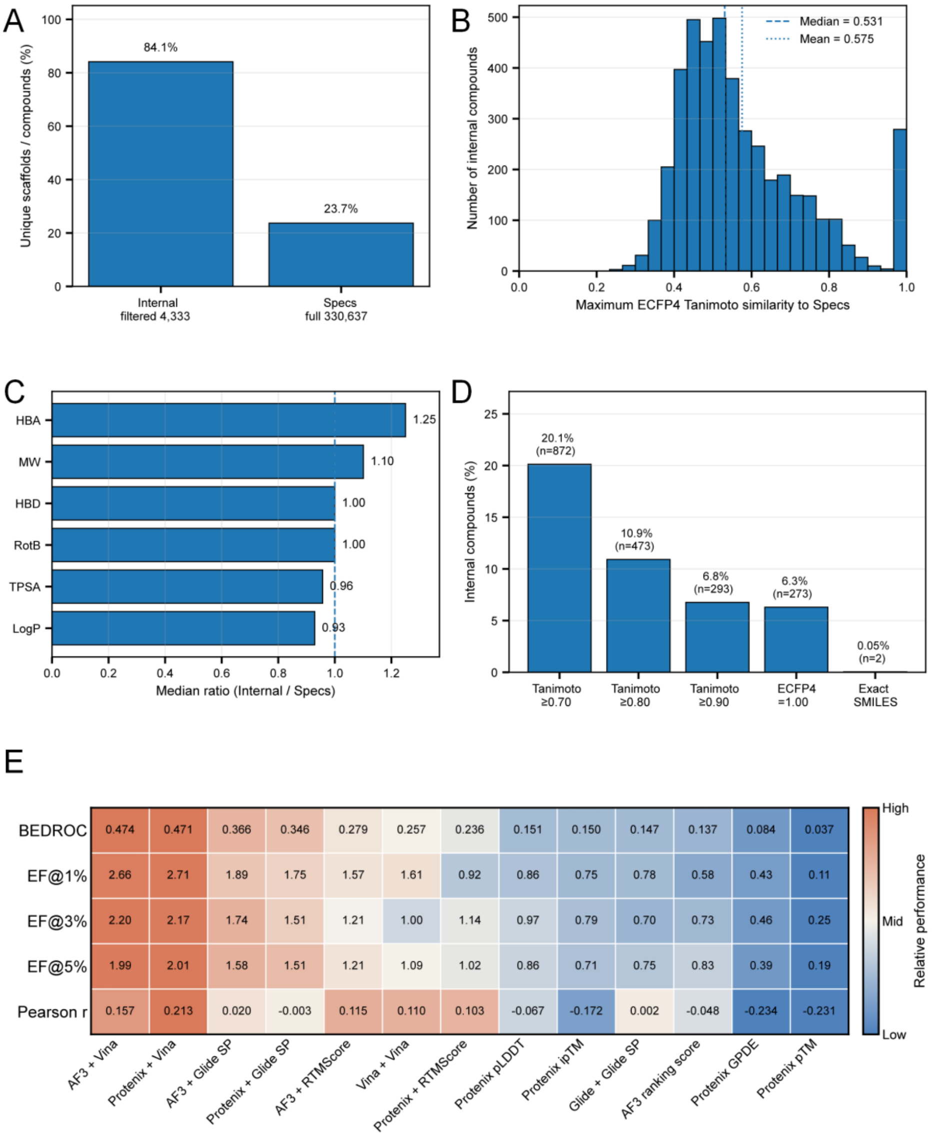

Figure S1. Chemical-space comparison and retrospective workflow evaluation. A, Bemis–Murcko scaffold diversity of the internal benchmark set and the Specs library. B, Maximum ECFP4 Tanimoto similarity from each internal compound to its nearest neighbor in the Specs library. C, Median ratios of representative physicochemical descriptors between the internal benchmark set and the Specs library. D, Fractions of internal compounds with Specs nearest neighbors above selected Tanimoto similarity thresholds and exact SMILES overlap. E, Heatmap summary of retrospective performance across 13 co-folding/docking–scoring pipelines, including Vina+Vina. BEDROC was the primary selection metric; EF values and Pearson r were complementary descriptors. The AF3 confidence metric is the composite ranking score.

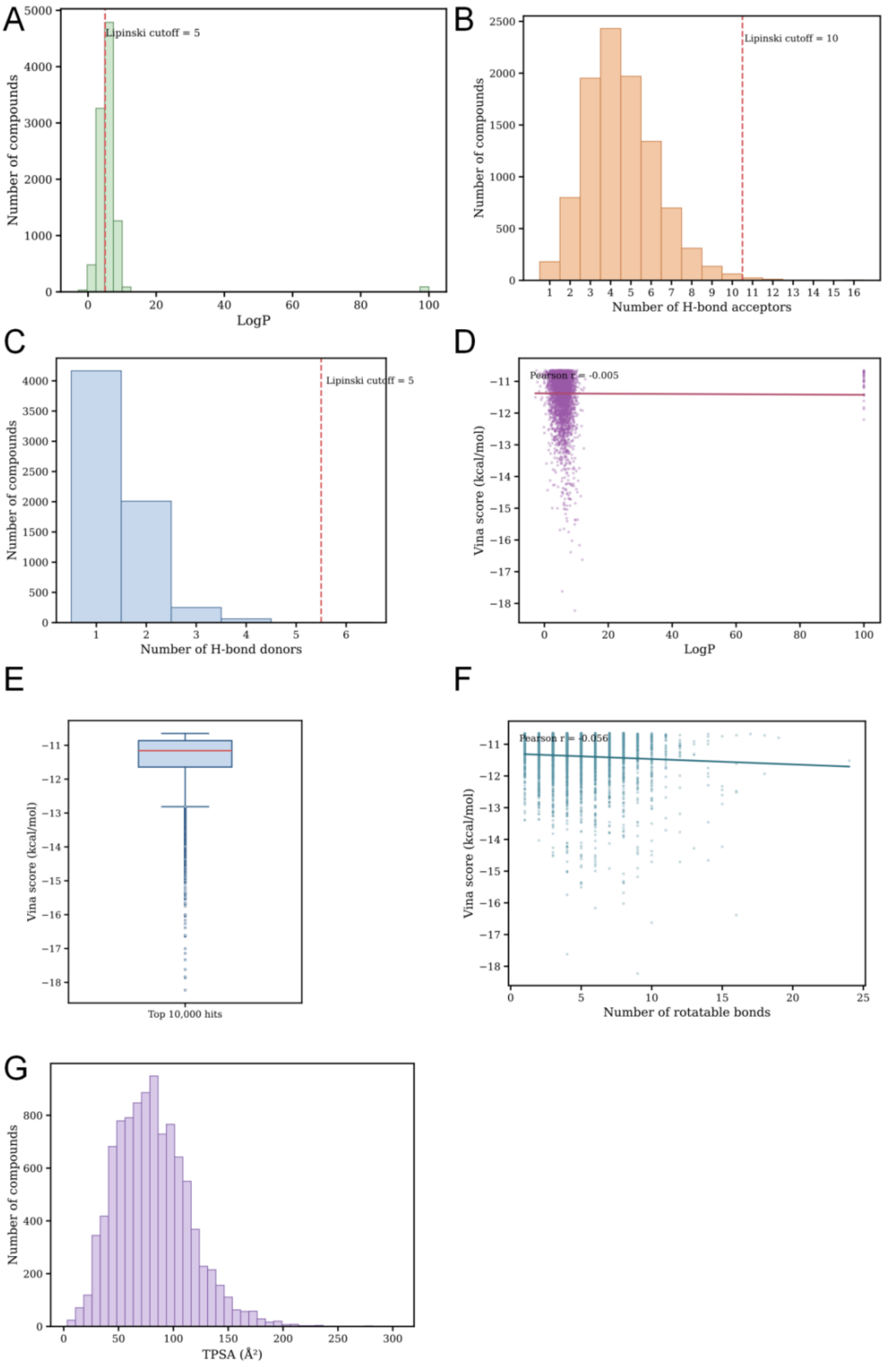

Figure S2. Physicochemical characterization of the top 10,000 compounds from the Specs screen. A, LogP distribution. B, Hydrogen-bond acceptor count distribution. C, Hydrogen-bond donor count distribution. D, Relationship between LogP and Vina score. E, Vina score distribution. F, Relationship between rotatable bond count and Vina score. G, TPSA distribution.

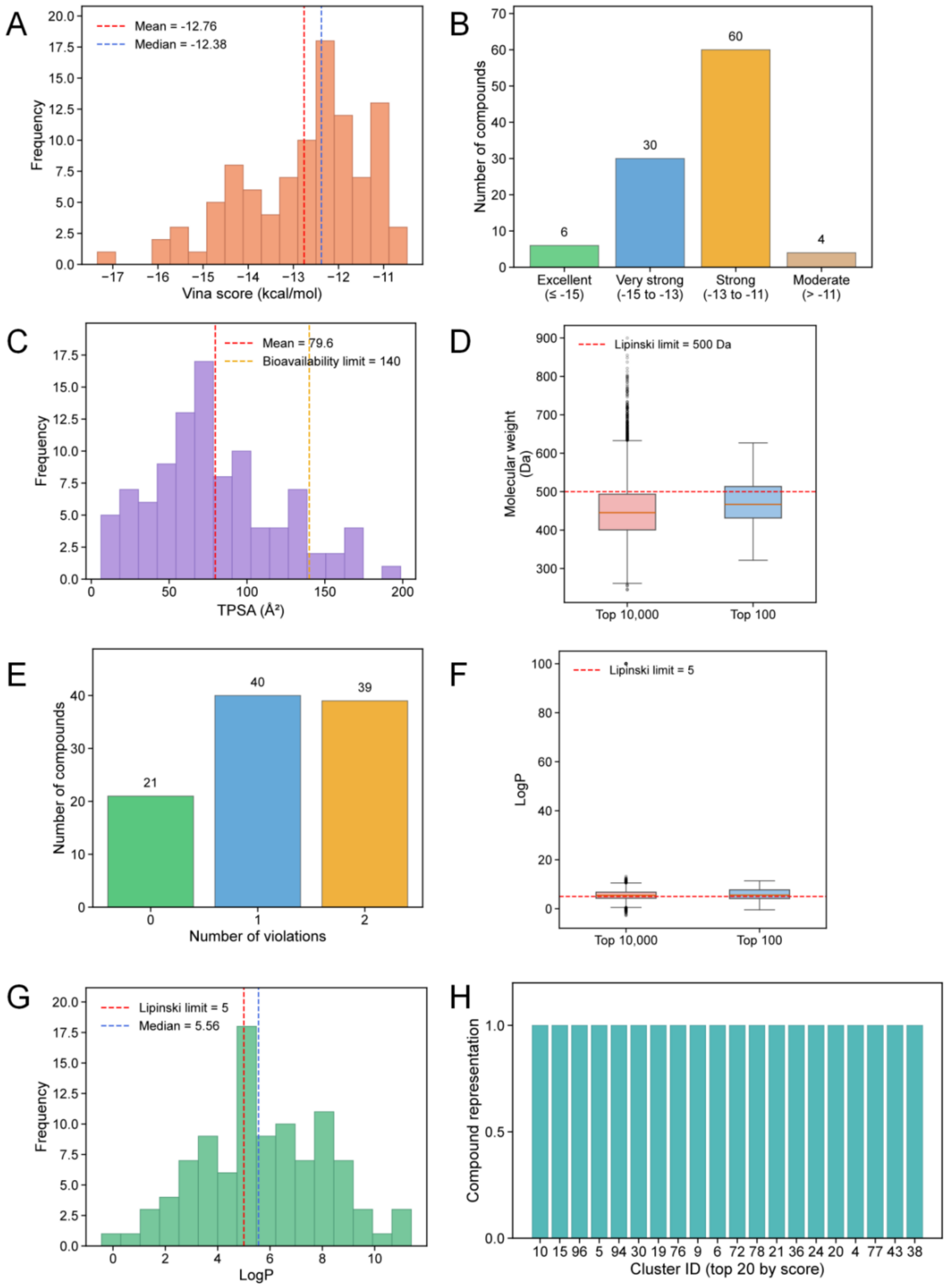

Figure S3. Additional characterization of the Top 100 cluster representatives. A, Vina score distribution. B, Docking-score category distribution. C, TPSA distribution. D, Molecular-weight comparison between the top 10,000 compounds and the Top 100 representatives. E, Lipinski-rule violation distribution. F, LogP comparison between the top 10,000 compounds and the Top 100 representatives. G, LogP distribution of the Top 100 representatives. H, Cluster representation of selected Top 100 compounds.

Supplementary Methods

Section S1. Receptor alignment and RMSD analysis

Receptors were prepared as described in Methods 4.4. For each available AF3 or Protenix complex selected by Vina in the retrospective benchmark, the receptor was superposed on the crystallographic TYK2 kinase domain using the common Cα atoms. Global Cα RMSD was calculated over these matched atoms. Pocket residues were defined by a receptor heavy atom within 8 Å of the bound reference ligand; pocket Cα RMSD was calculated after the same global fit, without a separate pocket alignment. The same procedure was applied to the receptor models associated with FV13 and TX14 after confirming the compound identities. Table S2 summarizes these comparisons.

Section S2. Conventional Vina docking control

Ligands were generated from SMILES with RDKit ETKDGv3, optimized with UFF for up to 500 iterations, and prepared with Open Babel and MGLTools. AutoDock Vina 1.2.7 used a fixed crystal receptor after removal of waters and the bound ligand. A 30 Å cubic search region covered the ATP-binding site, with exhaustiveness 32 and seed 2025. Up to five returned poses were evaluated in score-only mode, retaining the lowest score. Crystal and predicted receptors differ in unresolved regions and phosphorylation state, so this control compares complete workflows.

Section sS3. Enrichment and parental-cell sensitivity analyses

All 13 workflows followed the original score-processing procedure. Rows with missing scores or activity values were excluded before selecting the best available score per compound. Score directions were standardized so that higher values ranked first. For BEDROC, tied scores retained compound-ID order, and values were computed with RDKit at $\alpha = 80.5$. The parental-cell comparison used the labels defined in Methods 4.5 and retained both label classes within the 222-compound cohort. The 40% viability cutoff was an operational analysis definition, not a validated non-cytotoxicity threshold.

Table S1. BEDROC comparison of the 13 workflows.

Workflow / ranking metric	Original BEDROC	Parental-cell BEDROC	Parental-cell rank
AF3 + Vina	0.474	0.918	1
Protenix + Vina	0.471	0.786	6
AF3 + Glide SP	0.366	0.785	7
Protenix + Glide SP	0.346	0.802	5
AF3 + RTMScore	0.279	0.460	11
Vina + Vina	0.257	0.672	10
Protenix + RTMScore	0.236	0.304	12
Protenix pLDDT	0.151	0.913	2
Protenix ipTM	0.150	0.818	4
Glide + Glide SP	0.147	0.676	9
AF3 ipTM	0.137	0.283	13
Protenix GPDE	0.084	0.834	3
Protenix pTM	0.037	0.692	8

BEDROC uses $\alpha = 80.5$. The parental-cell cohort contains 222 follow-up compounds: 170 meet the activity and ≥40% viability criteria and are positive; 52 have viability <40% and are negative. Each pipeline uses its available scores as defined in Methods 4.5; rank 1 denotes the highest BEDROC. AF3+Vina ranks first in both analyses.

Table S2. Predicted receptor alignment to the crystallographic TYK2 kinase domain.

Retrospective receptors	Median global Cα RMSD (Å)	Median pocket Cα RMSD (Å)
AF3 + Vina	0.74	0.49
Protenix + Vina	0.57	0.89
**Prospective hit receptor**	**Global Cα RMSD (Å)**	**Pocket Cα RMSD (Å)**
FV13	0.90	0.65
TX14	0.93	0.67

Pocket RMSDs use the global receptor alignment and the pocket definition in Supplementary Methods S1. Retrospective values are medians across the available receptor models; FV13 and TX14 values describe the corresponding prospective models. Receptor similarity does not independently validate ligand poses.
